# RpoS role in antibiotic resistance, tolerance and persistence in *E. coli* natural isolates

**DOI:** 10.1186/s12866-024-03222-7

**Published:** 2024-03-05

**Authors:** Estela Ynés Valencia, Felipe de Moraes Gomes, Katia Ospino, Beny Spira

**Affiliations:** Departamento de Microbiologia, Instituto de Ciências Biomédicas, Universidade de São Paulo, São Paulo, SP Brazil

**Keywords:** RpoS, Antibiotic resistance, Tolerance, Persistence, *Escherichia coli*

## Abstract

**Background:**

The intrinsic concentration of RpoS, the second most abundant sigma factor, varies widely across the *E. coli* species. Bacterial isolates that express high levels of RpoS display high resistance to environmental stresses, such as temperature, pH and osmolarity shifts, but are less nutritional competent, making them less capable of utilising alternative nutrient sources. The role of RpoS in antibiotic resistance and persistence in standard laboratory domesticated strains has been examined in several studies, most demonstrating a positive role for RpoS.

**Results:**

Using disk diffusion assays we examined bacterial resistance to 15 different antibiotics, including $\upbeta$-lactams (penicillins, monobactams, carbapenems and cephalosporins), aminoglycosides, quinolones and anti-folates, in a representative collection of 328 *E. coli* natural isolates displaying a continuum of different levels of RpoS. There was an overall trend that isolates with higher levels of RpoS were slightly more resistant to these antibiotics. In addition, the effect of RpoS on bacterial tolerance and persistence to 3 different antibiotics - ampicillin, ciprofloxacin and kanamycin was evaluated through time-kill curves. Again, there was a small beneficial effect of RpoS on tolerance and persistence to these antibiotics, but this difference was not statistically significant. Finally, a K-12 strain expressing high levels of RpoS was compared with its isogenic RpoS-null counterpart, and no significant effect of RpoS was found.

**Conclusion:**

Based on a representative collection of the species *E. coli*, RpoS was found to have a very small impact on antibiotic resistance, tolerance, or persistence.

**Supplementary Information:**

The online version contains supplementary material available at 10.1186/s12866-024-03222-7.

## Introduction

RpoS ($\upsigma$^S^) is considered the second most important sigma factor in *E. coli*, controlling the transcription of genes associated with bacterial survival in stressful conditions and during the stationary phase [1, 2]. However, despite its importance, the concentration of RpoS across the *E. coli* species is not constant. RpoS expression and function are regulated at several levels (transcription, translation, post-translation and protein stability) and governed by several different inputs, such as regulatory proteins and small RNAs and the alarmone (p)ppGpp, which are themselves regulated by a multitude of other factors [3]. By tinkering with these factors evolution shapes the intrinsic levels of RpoS in a bacterial lineage [3, 4]. Over time, selective pressures acting on a population set the balance between self preservation (resistance to environmental stresses, positively influenced by RpoS) and nutritional competence (ability to use alternative nutrient sources, affected negatively by RpoS) [1, 5, 6]. The selection of null or attenuating mutations in *rpoS* has been demonstrated under different conditions of nutrient limitation and bacterial competition [7–11]. Conversely, under stressful conditions, that require the expression of genes related to bacterial protection and survival, bacteria with high levels of RpoS should predominate.

Several studies have reported the effect of RpoS on bacterial resistance to antibiotics (reviewed in [12]). For instance, an *rpoS*-negative K-12 strain was more sensitive to cephalosporin C, cephalexin, cephalothin, cephamicin C, trimethoprim and 1,2-benzisothiazolin-3-one than its *rpoS*^+^ counterpart [13]. Similarly, knockout of *rpoS* reduced the minimum inhibitory concentration (MIC) of the K-12 strain MG1655 against chloramphenicol, rifampicin and erythromycin [14]. On the other hand, it has recently been reported that knockout of *rpoS* did not affect antimicrobial resistance in an extended-spectrum $\upbeta$-lactamase (ESBL)-hypervirulent *Klebsiella pneumoniae* strain [15]. In *P. aeruginosa*, the *rpoS* null mutant displayed a much lower survival rate when exposed to biapenem, imipenem (in the stationary phase) and ofloxacin, despite having similar MIC values to that of the wild-type strain [16].

RpoS was also found to be important to antibiotic tolerance and persistence [16–18]. Tolerance of *P. aeruginosa* to ofloxacin was shown to be dependent on the presence of *rpoS* [19]. Additionally, subinhibitory concentrations of ampicillin resulted in increased mutagenesis and high frequency of mutants resistant to tetracycline, fosfomycin and rifampicin in *E. coli*, *P. aeruginosa* and *V. cholerae*. The effect of ampicillin on mutant frequency was dependent on *rpoS* [20]. Subinhibitory concentrations of ciprofloxacin induce the SOS response, which, in conjunction with RpoS, increases ampicillin and rifampicin resistance by approximately twenty-fold [21]. RpoS also increases mutagenesis that results in ciprofloxacin resistance in *P. aeruginosa* by partially modulating *dinB* [22]. Conversely, deletion of *rpoS* dramatically increased persistence in the presence of ampicillin via overexpression of the MqsR toxin (of the TA system MqsR/MqsA), suggesting that bacteria defective in the general stress response more readily produce persister cells [23].

In the present study, we examined the relationship between intrinsic RpoS levels, in a collection of 328 *E. coli* natural isolates, and bacterial susceptibility to 15 different antibiotics. These strains have been isolated from a water stream heavily contaminated with faecal material and were shown to be phylogenetically representative of the *E. coli* species, containing bacteria from most known phylogenetic groups - A, B1, B2, C, D, E and F [24]. We have found a great variability in RpoS levels in these isolates (ranging from 0 to 2.5 relative units with a continuum of ratios between these extremes) [24]. In light of previous studies linking RpoS to antibiotic resistance, tolerance and persistence, our aim was to determine whether RpoS affects antibiotic susceptibility in a collection of representative *E. coli* natural isolates. Another objective of this study was to screen for the presence of ESBL producers [25] in this collection. Though no statistically significant correlation between RpoS level and antibiotic sensitivity across the entire population has been observed, high RpoS strains tended to be less susceptible to some antibiotics when compared to strains with low RpoS levels. We also tested the tolerance and persistence towards the antibiotics ampicillin, ciprofloxacin and kanamycin, but found no significant difference between high and low RpoS strains.

## Material and methods

### Bacterial strains and growth conditions

We used a collection of 328 *E. coli* natural isolates from the Pirajuçara stream in São Paulo-SP, Brazil [24]. MC4100 [26] and its isogenic *rpoS*-negative isolate BS1154 (rpoS $\Delta$668A) [11] were used as positive and negative controls, respectively. Bacteria were always grown at 37^◦^ in lysogeny broth (LB) [27] and spread on either L-agar or Mueller-Hinton Agar plates.

### Antibiogram

To characterise the microbial resistance pattern of the isolates we used the Kirby-Bauer disk diffusion method, in which 15 commercial filter disks (CECON São Paulo, Brazil), each with a different antibiotic or combination of antibiotics were used as follows: Clavulanic Acid + Amoxicillin (AMC, 30 $\upmu$g); Amikacin (AMI, 30 $\upmu$g); Nalidixic Acid (NAL, 30 $\upmu$g) Imipenem (IMP, 10 $\upmu$g); Aztreonam (ATM, 30 $\upmu$g); Ceftazidime (CAZ, 30 $\upmu$g); Cefoxitine (CFO, 30 $\upmu$g); Cefotaxime (CTX, 30 $\upmu$g); Ceftriaxone (CRO, 30 $\upmu$g); Ciprofloxacin (CIP, 5 $\upmu$g); Cefepime (CPM, 30 $\upmu$g); Ertapenem (ETP, 10 $\upmu$g); Sulfamethoxazole + Trimethoprim (SUT, 25 $\upmu$g); Gentamicin (GM, 10 $\upmu$g); Meropenem (MER, 10 $\upmu$g). The disks were placed at an appropriate distance from each other on Mueller-Hinton agar plates, previously spread, with the help of a cotton swab, with a bacterial suspension of 0.5 McFarland turbidity standard, followed by incubation for 24 h at 37^◦^C. Susceptibility and resistance to each antibiotic was determined following the Clinical and Laboratory Standards Institute (CLSI) [28]. We measured the diameter of the inhibition halos as described by [29] with the help of the ImageJ software [30] by gauging the halo areas and converting to diameter sizes. We found that directly assessing diameters was less accurate than measuring areas.

Additionally, the disc-diffusion plates were also used to test for the presence of an ESBL-phenotype by using the Modified Double Disc Synergy Test as described [31].

### Minimum inhibitory concentration (MIC)

The MIC of each antibiotic - ampicillin, ciprofloxacin and kanamycin were assessed for each of the following bacterial strains: A05, E11, F19, G03, G20, I04, I12, J13, MC4100 and BS1154 in triplicates according to the CLSI directions [28]. Bacteria grown in LB medium were diluted 100-fold in medium Mueller Hinton and grown until an OD_600_ of $\sim 0.1$. Then $10^5$ bacteria/ml from each culture were added to each well of a 96-well plate containing serial dilutions of each antibiotic in medium Mueller Hinton and further grown for another 24 h. Turbidity was then evaluated in a microplate spectrophotometer (BioTek). The MIC was established by determining the lowest antibiotic concentration that prevented the growth of bacteria in the plate well.

### Killing curves

For the killing curves isolates A05, E11, F19, G03, G20, I04, I12, I25, J13 and J24 were used as well as the K-12 strains MC4100 and BS1154. We conducted the killing curves essentially as described [32]. Briefly, bacteria grown for 16 h in LB medium were diluted 100-fold in Mueller Hinton medium and grown until an OD_600_ of $\sim 0.5$. $10^8$ bacteria were inoculated in fresh Mueller Hinton containing 20X MIC of either ampicillin or ciprofloxacin or 10X MIC of kanamycin. All cultures were incubated for 240 minutes with agitation and samples were withdrawn every 15 min for kanamycin and every 30 min for ampicillin and ciprofloxacin-treated cultures, diluted when necessary with 0.9% NaCl and plated for assessing bacterial survival. Each assay was performed with at least 3 biological replicates. The MDK_99_ and MDK_99.99_ were determined from exponential regressions of the time-kill curves.

### Statistical analysis

Statistical analyses - bivariate regressions and Students’ t-tests were performed using JASP [33]. Pearson’s and Spearman’s coefficients were obtained for all regression analyses. Shapiro-Wilk analyses, also performed in JASP, showed that most variables were not normally distributed.

## Results

### Antibiograms of 328 *E. coli* natural isolates

We first applied the Kirby-Bauer disk diffusion assay to determine the susceptibility of the isolates to 15 different antibiotics: the aminopenicillin Amoxicillin in combination with Clavulanic acid (AMC), the monobactam Aztreonam (ATM); the carbapenems Ertapenem (ETP), Imipenem (IMP) and Meropenem (MER); the cephalosporins Ceftriaxone (CRO), Ceftazidime (CAZ), Cefepime (CPM), Cefoxitin (CFO) and Cefotaxime (CTX); the aminoglycosides Amikacin (AMI) and Gentamicin (GM); the quinolones Ciprofloxacin (CIP) and Nalidixic acid (NAL); and the anti-folates Sulfamethoxazol + Trimethoprim (SXT). A representative image of a disk diffusion plate is shown in supplementary Fig. S1 while supplementary Table S1 lists the results from all disk diffusion assays.

**Fig. 1:**
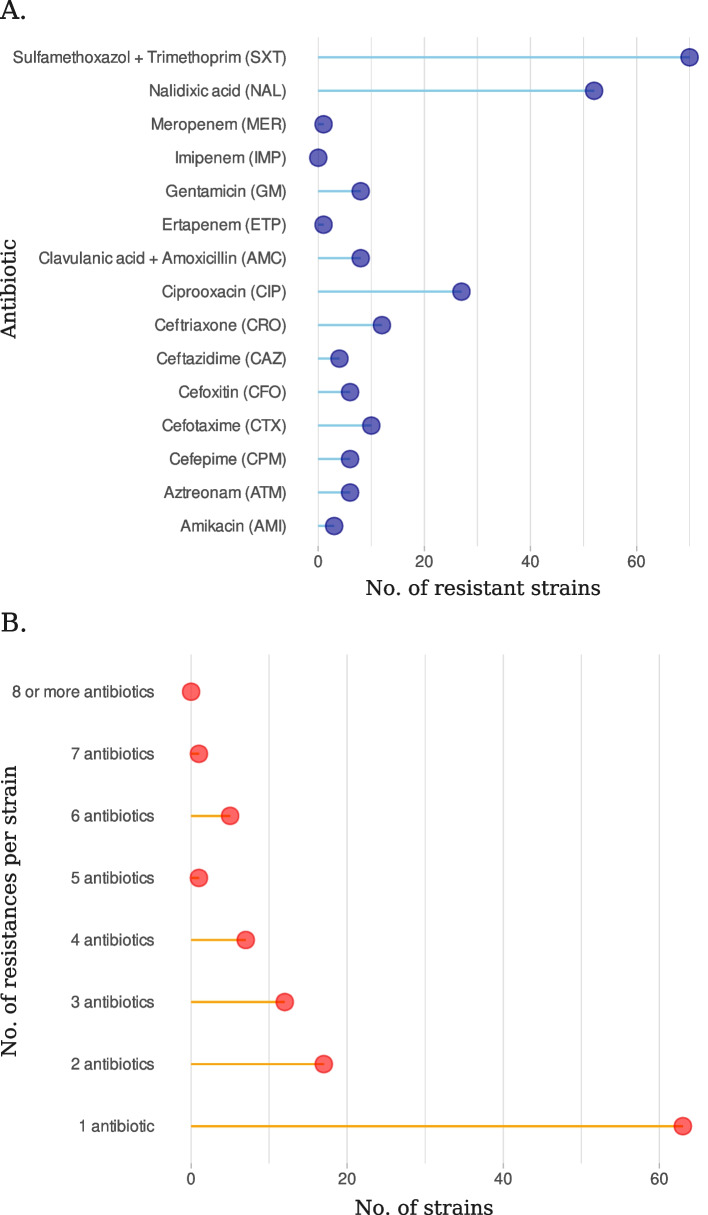
**A** Number of isolates with resistance to each tested antibiotic. The halo diameters in antibiograms to 15 different antibiotics were assessed and used to determine the resistance status of the bacteria. **B** Distribution of the number of resistances per strain

A total of 106 isolates (32% of 328) were resistant to at least one antibiotic (Fig. 1A and B). No strain was resistant to imipenem and 70 isolates displayed resistance to Sulfamethoxazol + Trimethoprim, the less effective antibiotic treatment in this set of strains. Sixty three isolates were resistant to a single antibiotic, while strain L25 displayed resistance to 7 different antibiotics, the highest level of multi-resistance in this collection. Forty-three isolates showed an intermediate number of resistances (2-6 different resistances) (Fig. 1B). In addition, 6 strains (1,8% of 328) were shown to be ESBL producers - B12, G29, J11, L15, L19 and L25. Figure S2 in the supplement shows the isolates displaying the ESBL phenotype in a double-disk synergy test.

### Effect of RpoS on antibiotic resistance in natural isolates

Our principal aim was to find out whether RpoS contributes to antibiotic resistance in *E. coli* natural isolates (described in detail in [24]). With that in mind, we correlated the diameters of the antibiogram halos of each isolate (response variable) with their corresponding RpoS level (predictor) (Table 1). Neither variable - RpoS concentration or halo diameter is normally distributed (Shapiro-Wilk test with *p*<0.001), thus Spearman correlation coefficients, in addition to Pearson coefficients, were included in the analysis. Overall, the correlation coefficients were very low and without statistical significance. The exceptions were CPM [$r = -0.146\ (p = 0.02); \rho = -0.128\ (p = 0.02)$] and SXT [$\rho = -0.122\ (p = 0.042)$] that showed low but statistically significant correlations ($p < 0,05$) with RpoS. In both cases RpoS level was negatively associated with the halo diameter, suggesting a beneficial effect of RpoS on the susceptibility to these antibiotics. It is worth noting that, as a general rule, susceptibility to one antibiotic was often associated with susceptibility to others, i.e., the change in halo diameters largely correlated among the different antibiotics. For instance, susceptibility to amikacin was not only associated with susceptibility to gentamycin, another aminoglycoside ($r = 0.470, \rho = 0.780; p < 0.001$), but also to a less extent with other categories of antibiotics (Table S3 in the supplement).

**Table 1:** Correlation between RpoS and antibiotic susceptibility

		Pearson		Spearman	
Predictor	Response variable	r	*p*	$\rho$	*p*
RpoS	AMC	$-0.078$	0.161	$-0.099$	0.073
	AMI	0.015	0.788	$-0.006$	0.908
	ATM	$-0.049$	0.379	$-0.046$	0.402
	CAZ	$-0.087$	0.115	$-0.042$	0.446
	CFO	0.017	0.752	$-0.015$	0.786
	CIP	0.049	0.377	$-0.004$	0.935
	CPM	$$-0.146^{**}$$	0.008	$$-0.128^*$$	0.020
	CRO	$-0.083$	0.135	$-0.068$	0.217
	CTX	$-0.090$	0.103	$-0.031$	0.579
	ETP	$-0.026$	0.645	$-0.037$	0.509
	GM	0.017	0.755	$-0.026$	0.642
	IMP	$-0.092$	0.098	$-0.091$	0.101
	MER	$-0.089$	0.106	$-0.070$	0.206
	NAL	0.055	0.319	0.007	0.897
	SXT	$-0.054$	0.330	$-0.118^*$	0.032

* p < .05, ** p < .01

Most isolates in the collection express intermediate levels of RpoS (mean RpoS level = 0.91 ± 0.41 arbitrary units [24]). As a result, small differences in RpoS content may not disclose whether this sigma factor has a significant effect on antibiotic susceptibility. In order to refine our analysis, we focused on strains that express the highest and lowest levels of RpoS. A group of 41 strains expressing more than one standard deviation of RpoS (RpoS $\ge 1.33$), was compared to another group of isolates representing 51 strains expressing less than one standard deviation of RpoS (RpoS $\le 0.5$). Table 2 shows that the two RpoS groups displayed significant differences regarding sensitivity to the antibiotics AMC, CPM, CRO, CTX and SXT. For the sake of comparison, it can be seen that the two groups also differed in their sensitivity to acid and dehydration stresses, as expected (Table S2 in the supplement shows the full dataset of bacterial survival to these stresses).

**Table 2:** Antibiotic and stress sensitivity of high and low RpoS sets of strains

**Antibiotic**	**Halo – low RpoS**^1^	**Halo – high RpoS**^2^	***p***^5^
AMC	31.06	28.94	0.03
AMI	26.56	26.74	0.87
ATM	37.80	36.80	0.5
CAZ	33.68	32.87	0.42
CFO	29.30	29.60	0.78
CIP	36.63	36.73	0.95
CPM	38.41	36.00	0.005
CRO	37.1	35.19	0.04
CTX	37.37	35.47	0.04
ETP	37.02	36.69	0.62
GEN	24.85	24.78	0.93
IMP	35.04	33.46	0.06
MER	36.77	35.75	0.14
NAL	27.02	27.15	0.94
SXT	31.84	25.69	0.014
**Stress**	**Survival – low RpoS**^3^	**Survival – high RpoS**^4^	***p***^5^
Acid	44.69	64.56	0.03
Dehydration	13.13	38.94	0.00004

^1^ Mean halo diameter (in mm) of isolates with RpoS $\le 0.5$

^2^ Mean halo diameter (in mm) of isolates with RpoS $\ge 1.33$

^3^ Mean % survival of isolates with RpoS $\le 0.5$

^4^ Mean % survival of isolates with RpoS $\ge 1.33$

^5^ Students’ t-test *p*

### Effect of RpoS on antibiotic resistance in K-12 strains

**Fig. 2:**
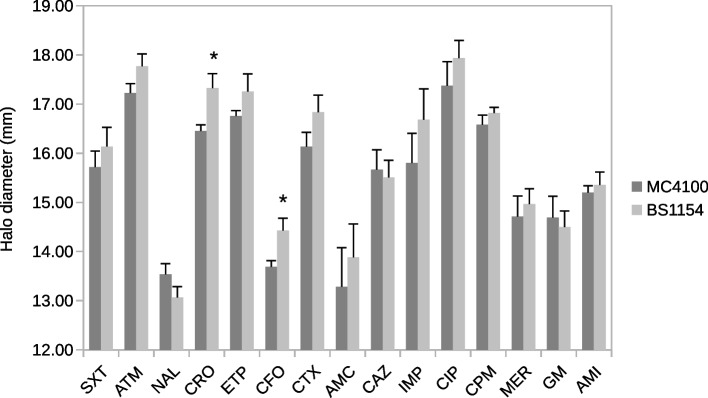
Sensitivity of MC4100 (high RpoS) and BS1154 (*rpoS* mutant) to antibiotics. Antibiograms were performed as described in Material and methods section. Each bar corresponds to the mean of six independent cultures. *, p < 0.05 according to t-Students’ test

Additionally, we assessed the effect of RpoS in the laboratory strain MC4100 [26] and in its *rpoS* spontaneous null mutant BS1154 [11]. MC4100 carries an *rssB* mutation that strongly elevates the level of RpoS. Figure 2 shows that the halos of inhibition of the *rpoS* mutant are in most cases slightly larger than those of the wild-type strain, but the difference between the strains was only significant for the cephalosporines cefoxitine (CFO) and ceftriaxone (CRO). It can thus be concluded that RpoS has a small effect on the susceptibility to some antibiotics in a high-RpoS *E. coli* K-12 strain and also across a population of *E. coli* natural isolates.

### Effect of RpoS on bacterial tolerance and persistence in selected strains

**Fig. 3:**
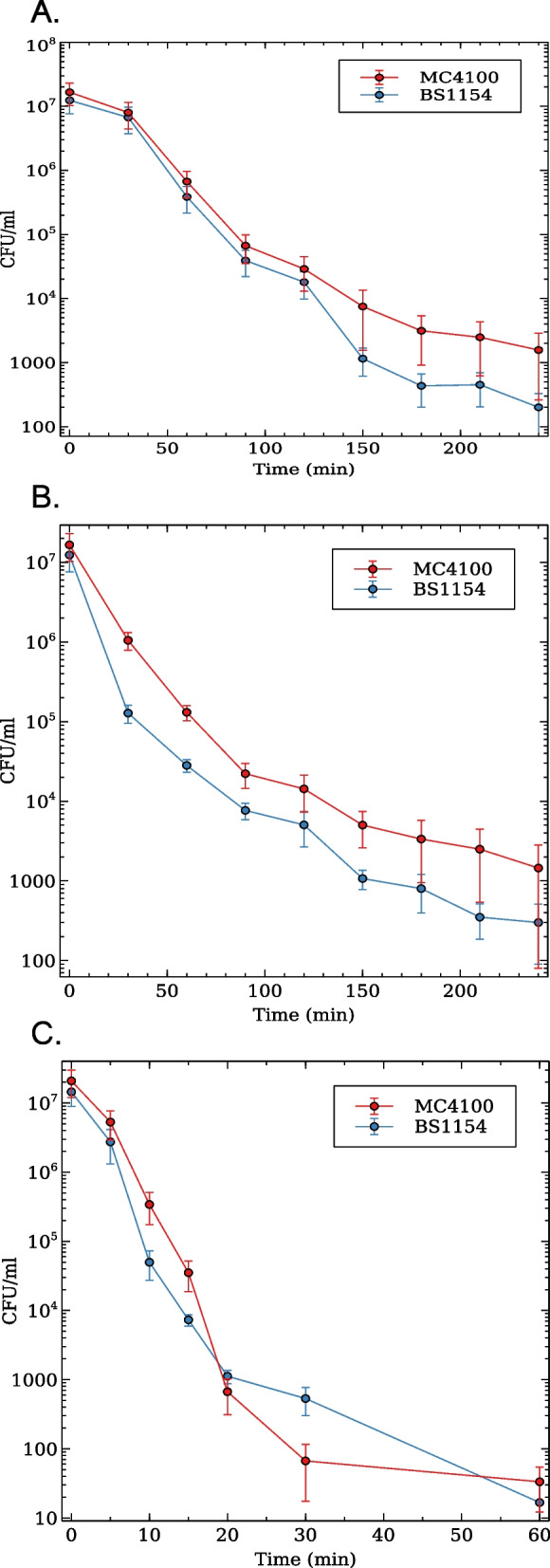
Killing curves of strains MC4100 (*rpoS*^+^) and BS1154 (*rpoS*^-^) subjected to high concentrations of ampicillin, ciprofloxacin or kanamycin. $10^7$ exponentially growing cells diluted in medium Mueller Hinton containing 20X MIC (ampicillin and ciprofloxacin) or 10X MIC in the case of kanamycin for each strain. Samples were withdrawn at time 0 and at different time-intervals, depending on the antibiotic treatment. Each point corresponds to the mean of at least three independent experiments ± standard error of the mean. Bacterial survival was assessed by colony counting on L-agar plates

It is important to note that while resistance to antibiotics is the most definitive response to these toxic molecules, bacteria can also defend themselves through two other processes: tolerance and persistence [34]. Both processes are transient and time-dependent, providing some degree of protection for a definite period of time. Tolerant bacteria can survive, but not grow in the presence of a high concentration of an antibiotic and resume growth once the antibiotic is removed. In contrast, persistence is associated with the existence of a heterogeneous population, in which only a fraction of the population is tolerant to antibiotics. Importantly, both tolerance and persistence facilitates the evolution of resistance [35]. To test whether RpoS affects tolerance or persistence to different classes of antibiotics - $\upbeta$-lactams, quinolones and aminoglycosides, we performed killing curves of selected isolates and of strains MC4100 and BS1154 (MC4100 *rpoS*) in the presence of a 20-fold MIC of ampicillin (40 $\upmu$g/ml) or ciprofloxacin (60 ng/ml) or a 10-fold MIC of kanamycin (10 $\upmu$g/ml). Figure 3 shows that both MC4100 and BS1154 strains showed very similar biphasic killing curves.

Tolerance and persistence are, respectively, assessed by calculating the MDK_99_ and MDK_99.99_ (minimum duration of time needed to kill 99% and 99.99% of the population) obtained in time kill curves [34]. It has been stipulated that tolerant cells display an increase in MDK_99_, while persistent cells show similar MDK_99_ values but higher MDK_99.99_. Approximated exponential regressions of the time kill curves showed that both strains displayed very similar MDK_99_ and MDK_99.99_ values under the three antibiotic treatments (Table 3). These results suggest that RpoS does not play a significant role in tolerance or persistence to ampicillin, ciprofloxacin or kanamycin in this high-RpoS K-12 strain.

**Table 3:** Minimal duration of killing of strains MC4100 and its isogenic *rpoS* mutant and of natural isolates with different RpoS levels. The MDK_99_ and MDK_99.99_ were calculated from time-kill curves depicted in Fig. 3

Strain	RpoS level	MDK	Ampicillin	Ciprofloxacin	Kanamycin
MC4100	2.5^a^	MDK_99_	102.57	97.77	103.95
		MDK_99.99_	205.13	195.55	207.91
BS1154	0	MDK_99_	93.03	82.03	107.85
		MDK_99.99_	186.07	164.06	215.70
J24	0	MDK_99_	ND^b^	82.09	39.70
		MDK_99.99_		164.18	79.40
J13	0.24	MDK_99_	133.48	54.89	22.36
		MDK_99.99_	266.97	109.78	44.71
G20	0.26	MDK_99_	122.48	37.75	21.32
		MDK_99.99_	244.96	75.49	42.64
F19	0.28	MDK_99_	95.15	36.26	12.25
		MDK_99.99_	190.30	72.52	24.50
G03	0.29	MDK_99_	120.55	66.74	12.86
		MDK_99.99_	241.11	133.48	25.73
A05	1.72	MDK_99_	116.88	47.77	7.94
		MDK_99.99_	233.76	95.54	15.88
I04	1.79	MDK_99_	104.43	18.42	26.62
		MDK_99.99_	208.85	36.84	53.24
E11	1.99	MDK_99_	100.33	88.56	26.62
		MDK_99.99_	200.66	177.12	53.24
I12	2.22	MDK_99_	137.78	115.13	28.08
		MDK_99.99_	275.76	230.26	56.16
I25	2.48	MDK_99_	109.65	65.79	20.74
		MDK_99.99_	219.29	131.58	41.49

^a^ RpoS data for MC4100 and BS1154 are from [36], all others are from [24]

^b^ strain resistant to ampicillin

We also evaluated the MDK of some *E. coli* natural isolates at both extremes of the RpoS spectrum (Table 3). As with MC4100 and its *rpoS* isogenic strain, the MIC for ampicillin, ciprofloxacin and kanamycin was assessed in each isolate (supplementary Table S4) and bacteria survival were evaluated in time-kill curves. On average, the time-kill curves showed that the high-RpoS strains - A05, I04, E11, I12, I25 were slightly more tolerant to kanamycin and marginally more susceptible to ampicillin than those of the low-RpoS group (J24, J13, G20, F19 and G03). The mean MDK_99_ of the high and low-Rpos groups regarding ampicillin, ciprofloxacin and kanamycin respectively were 113.8, 67.1 and 22.0 min (high-RpoS) against 117.9, 55.5 and 21.7 min (low-RpoS strains). t-test analyses revealed no significant difference between high and low-RpoS strains for any antibiotic. It should be noticed, however, that strain J24 (RpoS = 0) is resistant to ampicillin, and was not included in the average calculation for this antibiotic. The time-kill curves and MDK assessments confirm that RpoS has at most a marginal effect on antibiotic tolerance in *E. coli*.

## Discussion

Many traits influence the level of resistance or heteroresistance to antibiotics, RpoS is allegedly one of them [12]. Previous studies have used isogenic laboratory strains displaying *rpoS*^+^ and *rpoS*^-^ phenotypes to explore this subject. The principal novelty of the present study is twofold: (1) the use of natural isolates in addition to laboratory strains and (2) not been limited to confronting an RpoS^0^ mutant against an RpoS^+^ strain, but we also examined a range of different strains with a continuum of RpoS intrinsic levels [4, 24]. Based on the majority of previous reports [12–14] our expectation was that RpoS would have a positive effect on bacterial resistance or tolerance towards antibiotics. However, we found only a small or marginal effect on antibiotic resistance and almost no effect on tolerance and persistence.

It has been shown that, in addition to their specific mechanism of action, different classes of antibiotics elicit the production of reactive oxygen species (ROS) that can damage key cellular components [37] and that subinhibitory concentrations of $\upbeta$-lactam antibiotics induce the RpoS regulon [20]. RpoS coordinates the general stress response, which, among other things, provide protection against oxidative stress [38]. Once RpoS is induced by antibiotics, we would expect that it would provide protection to ROS and increase cell survival, but this is not what the kill curves showed. Possibily, the high concentrations of bactericidal antibiotics used in our assays were too harsh to induce any meaningful effects on RpoS expression or stability. However, tolerance and persistence are, by definition, tested against high antibiotic concentrations, therefore, if subinhibitory antibiotic concentrations are able to elicit RpoS protection, this is an entirely different phenomenon. It should be noticed that although the time-kill curves were started with bacteria in mid-exponential phase (OD_600_ $\sim 0.5$), very similar kill curves were obtained with stationary-phase bacteria (OD_600_ $\sim 2.5$), i.e., they also failed to show an effect of RpoS on bacterial tolerance or persistence (supplementary Fig. S3).

Rami et al. [14] have shown that RpoS played a role in bacterial resistance (increase in MIC) in the *E. coli* wild-type strain MG1655 towards chloramphenicol, rifampicin and erythromycin. In our analysis, none of these three antimicrobials were tested, so we cannot exclude the possibility that our strains may respond differently to them. Evidence regarding whether RpoS influences tolerance and persistence is, however, conflicting. It has been demonstrated by [23] that deletion of *rpoS* dramatically increases persistence in *E. coli*, indicating that RpoS inhibits persister formation, whereas [39] found that deletion of *rpoS* increased persistence to ampicillin and norfloxacin but not gentamycin. In contrast, putrescine has been shown to enhance persistence by stimulating *rpoS* expression, while ectopic expression of *rpoS* in the absence of putrescine also induced persistence [40]. Conversely, N-starved *E. coli* *rpoS* knockout formed similar levels of persisters as the wild-type strain [18]. The discrepancies observed in the studies investigating RpoS and antibiotic responses may be explained by the fact that different experimental setups produce different results. These conflicting results suggest that RpoS may affect persistence through one or more factors that are still unknown. The genetic background of the strain may be relevant in this regard. Our study found that RpoS has no effect on both isogenic laboratory strains (the high-RpoS MC4100 strain and its isogenic *rpoS* mutant) and on 10 natural isolates with varying levels of RpoS and different genetic backgrounds.

The results of our study revealed that RpoS is weakly associated with resistance levels towards some antibiotics. However, not in all cases this effect was statistically significant. In addition, for most antibiotics the correlation with RpoS could be noticed only when the highest and lowest RpoS strains were directly confronted, i.e., after 68% of the strains with RpoS values within 1 SD were excluded. This indicates that the influence of RpoS on antibiotic resistance is characterised by a blurred threshold in which the majority of strains that express (close to) average levels of RpoS do not significantly differ in their RpoS-related susceptibility to antibiotics. Accordingly, it has been shown that the K-12 strain W3110 $\Delta$*rpoS* displayed 2-fold higher MICs for ampicillin and norfloxacin, a 2-fold lower MIC for gentamicin and no effect on the MIC for trimethoprim compared to the wild-type W3110 strain, indicating that the effect of RpoS on bacterial resistance is mild and the direction of the effect depends on the type of antibiotic [39].

Another interesting but not totally surprising result was that the degrees of susceptibility to different antibiotics were significantly associated with one another, indicating that, in general, strains with low susceptibility to one antibiotic were also less susceptible to other antibiotics, including those of different classes. It is evident from these results that intrinsic susceptibilities are not determined by acquired antibiotic-specific mechanisms as in the case of *bona fide* antibiotic resistance (plasmid-mediated resistance, enzymatic degradation and so on), but is governed by a more general system, such as the presence or absence of porins that provide restrictive permeability [41–43] or the presence of nonspecific efflux pumps [44].

In conclusion, we demonstrated that RpoS, the coordinator of the general stress response, is only weakly associated with bacterial resistance, tolerance or persistence to different classes of antibiotics.

### Supplementary Information

**Supplementary material 1.**

## Data Availability

No datasets were generated or analysed during the current study.
