# Supplementary material for: RpoS role in antibiotic resistance, tolerance and persistence in E. coli natural isolates
Source: BMC Microbiol. 2024 Mar 5;24:72. doi: 10.1186/s12866-024-03222-7 (PMC11323705; doi:10.1186/s12866-024-03222-7)
Supplement: Supplementary file 1 — Supplementary material 1. [file 12866_2024_3222_MOESM1_ESM.pdf]

RpoS role in antibiotic resistance, tolerance and persistence in *E. coli* natural isolates

Ynes Valencia[1], Felipe Moraes[1], Katia Ospino[1], and Beny Spira[1]*

[1]Departamento de Microbiologia, Instituto de Ciências Biomédicas
Universidade de São Paulo, São Paulo-SP, Brazil

*Corresponding author, email: benys@usp.br

SUPPLEMENTARY FILE

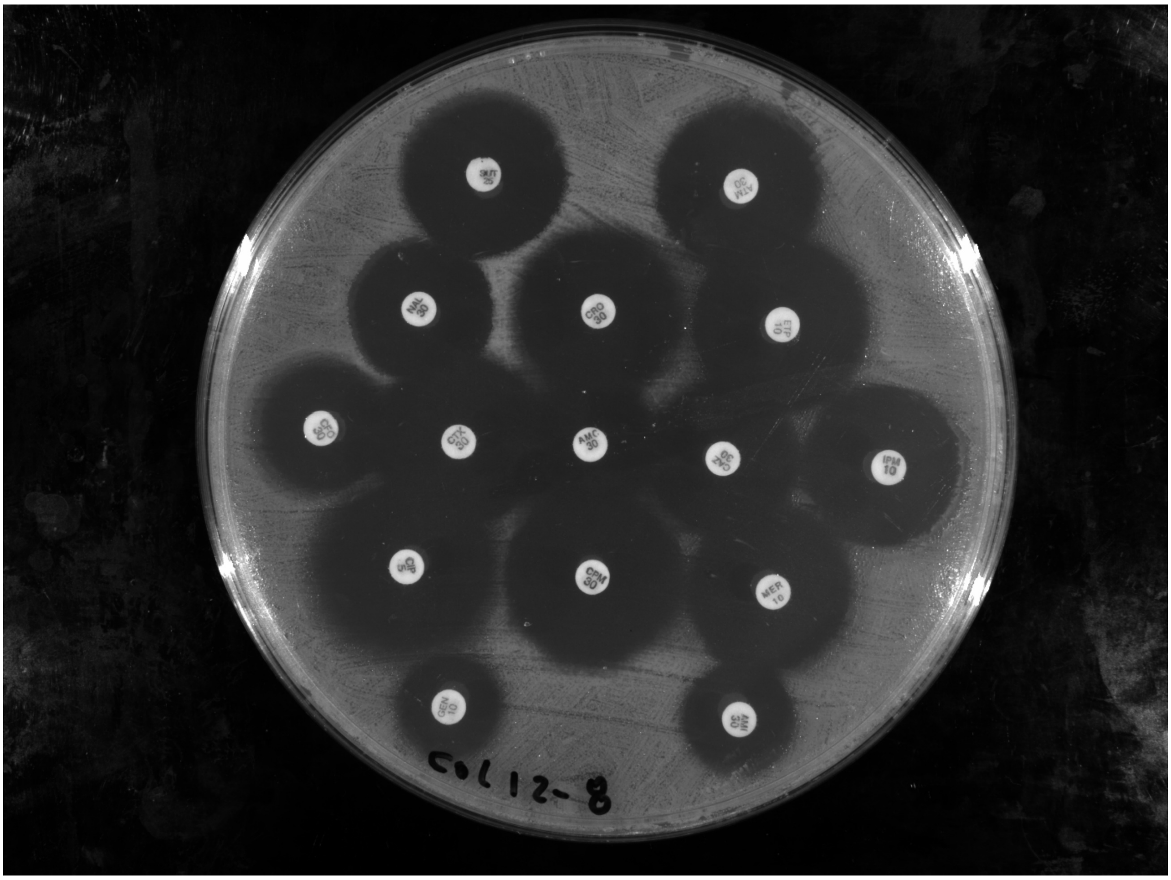

Fig S1. Disk diffusion assay of isolate K07

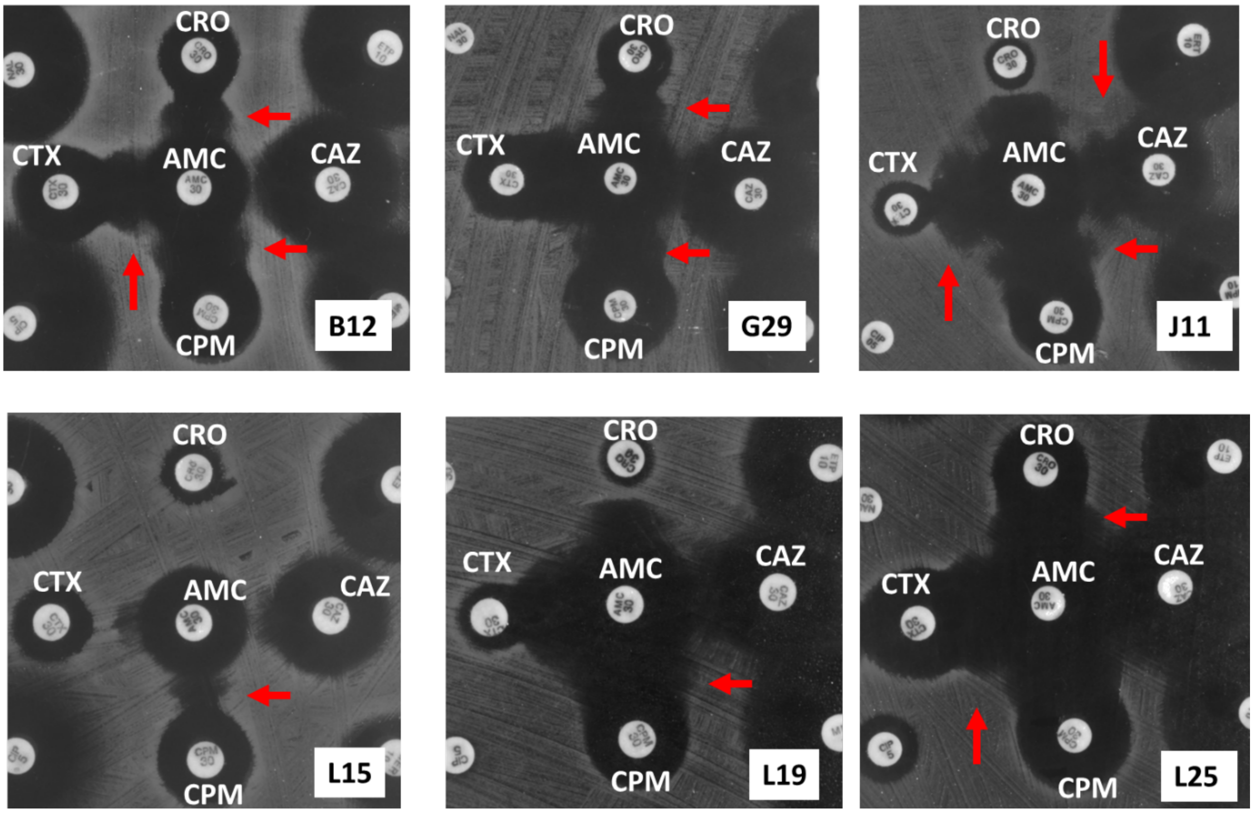

Fig S2. Antibiogram image of ESBL-producing of *E. coli* isolates B12, G29, J11, L15, L19 and L25 by double-disk synergy test. Amoxicillin-clavulanic acid (AMC); ceftriaxone (CRO); cefotaxim (CTX); ceftazidime (CAZ); cefepime (CPM).

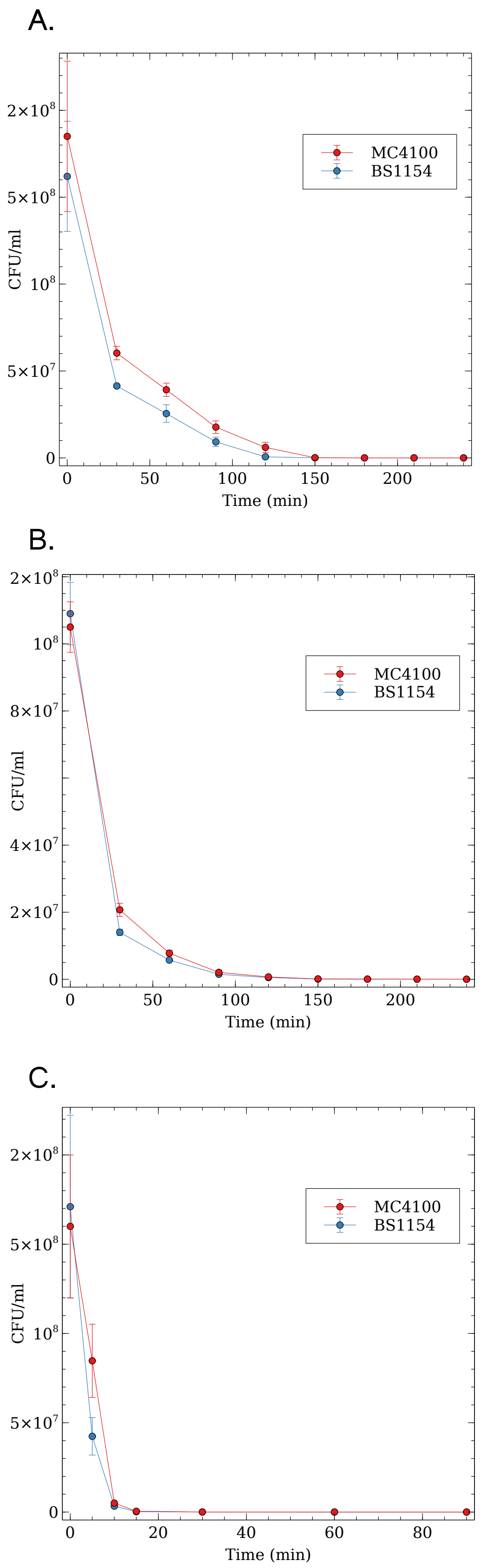

Fig S3. Killing curves of strains MC4100 (*rpoS*$^+$) and BS1154 (*rpoS*$^-$) harvested at the stationary phase (OD_{600} = 2.5) subjected to high concentrations of (A) ampicillin, (B) ciprofloxacin or (C) kanamycin. 10^8 exponentially growing cells diluted in medium Mueller Hinton containing 20X MIC (ampicillin and ciprofloxacin) or 10X MIC in the case of kanamycin for each strain. Samples were withdrawn at time 0 and at different time-intervals, depending on the antibiotic treatment. Each point corresponds to the mean of at least six independent cultures ± standard error of the mean. Bacterial survival was assessed by colony counting on L-agar plates.

Table S1. RpoS levels and halo diameters (in mm) of 328 natural isolates

	RpoS*	CRO	AMC	CAZ	CPM	CTX	ATM	CFO	ETP	IMP	MER	CIP	NAL	AMI	GM	SXT
A01	1.27	34.66	28.67	33.49	36.2	36.02	35.9	30.84	36.67	33.35	36.63	40.78	28.65	28.25	26.18	34.38
A02	1.31	37.66	35.19	36.27	38.56	38.78	40.58	30.22	36.17	33.38	37.57	44.42	31.52	28.25	25.82	37.08
A03	0.94	37.34	33.94	33.31	37.79	39.46	40.66	33.16	36.2	34.39	37.11	40.37	33.13	29.86	31.78	36.41
A04	0.96	34.31	28.82	32.57	36.23	34.9	35.75	28.08	37.45	31.81	37.59	34.9	22.97	28.82	26.59	32.53
A05	1.72	38.05	35.51	35.78	38.78	39.45	42.28	31.21	37.26	35.64	35.64	44.16	37.84	28.16	28.87	37.84
A06	0.72	35.55	28.92	33.89	36.76	38.2	40.35	31.52	36.62	32.44	35.11	44.1	31.16	29.86	27.98	33.8
A07	0.74	39.36	37.92	36.14	39.81	38.94	41.47	31.07	40.4	35.03	37.85	34.96	12.54	29.89	27.16	36.8
A08	0.92	33.13	27.25	31.92	34.07	34.93	35.9	28.34	35.19	30.26	34.14	37.56	27.98	26.5	25.96	33.81
A09	0.52	36.99	33.4	34.48	39.49	39.23	39.62	32.28	38.55	33.94	36.54	39.23	33	29.05	29.03	36.54
A10	1.01	35.14	30.76	35.73	37.26	41.02	37.17	30.52	37.35	34.89	39.9	42.86	32.67	29.5	27.44	32.1
A11	1.34	38.42	31.96	33.97	39.4	37.97	39.58	32.82	39.49	40.00	38.59	41.06	25.37	36.85	25.69	33.18
A12	0.93	36.04	31.12	34.01	37.87	37.73	38.42	31.21	37.83	32.77	36.31	45.18	32.23	28.25	27.75	35.78
A13	0.9	35.61	24.3	33.45	35.63	36.67	36	29.5	38.87	35.26	37.59	0.00	0.00	29.09	0.00	0.00
A14	0.86	38.01	29.59	35.02	39.15	39.32	39.68	33.67	39.68	35.68	38.28	42.67	33.08	29.22	27.58	0.00
A15	0.87	37.03	27.97	35.51	37.84	38.59	39.48	31.38	39.48	32.91	37.71	41.71	30.89	27.88	27.35	35.64
A16	0.89	36.54	27.59	33.4	37.19	36.99	35.06	31.38	36.59	33.44	36.67	39.81	29.28	28.07	26.03	33.9
A17	0.64	37.2	33.93	34.88	37.39	38.42	39.99	30.98	37.92	34.03	36.99	33.61	24.47	27.98	25.69	32.82
A18	0.73	35.36	30.53	33.63	36.36	37.09	39.92	30.85	37.98	33.44	36.85	43.47	31.66	27.57	25.82	35.55
A19	0.5	35.87	33.18	33.76	39.1	39.45	40.44	32.5	40.17	35.49	37.46	41.04	28.47	28.38	26.4	0.00
A20	0.94	32.91	29.24	32.95	36	35.55	36.88	29.1	37.12	32.33	36.38	38.2	30.66	26.67	25.15	0.00

A21	0.53	36.86	26.25	35.87	37.99	37.21	40.49	31.11	37.97	33.44	36.32	38.32	35.37	27.48	26.09	0.00
A22	1.34	36.58	32.64	35.09	37.52	38.28	39.9	29.55	39.9	35.27	38	45.17	33.81	27.66	10.74	0.00
A23	0.59	37.75	30.66	34.3	39.18	39.12	41.33	31.28	41.11	33.49	38.79	37.21	30.58	28.87	27.76	34.52
A24	0.84	35.18	29.39	34.05	36.84	37.8	37.36	29.69	37.36	32.6	35.79	38.5	28.9	25.37	24.81	32.87
A25	0.68	33.04	24.3	31.52	37.48	36.7	36.95	14.75	37.48	32.99	36.41	36.09	27.25	35.42	26.27	0.00
B01	1.08	33.85	27.8	32.28	34.21	33.81	33.22	25.55	34.07	30.8	33.89	33.85	26	24.06	21.69	28.7
B02	0.84	37.62	29.65	33.64	36.94	37.17	35.86	26.18	34.34	32.55	34.44	33.94	29.86	26.05	23.36	33.13
B03	1.16	36.44	29.23	30.89	35.15	35.22	35.94	26.36	34.79	30.71	32.32	38.51	29.63	22.86	20.85	34.25
B04	0.71	35.11	27.8	32.69	36.94	36.08	36.23	26.34	36.23	31.83	32.69	37.62	28.11	25.87	25.2	34.33
B05	0.59	34.52	31.07	32.73	35.24	32.81	36.54	26.22	37.88	34.48	34.92	35.68	31.33	26.11	24.21	39.54
B06	2.21	37.12	23.09	32.33	37.53	36.93	37.02	29	34.43	30.61	32.42	36.38	28.43	27.66	26.45	31.38
B07	1.62	36.32	26.55	30.93	36.81	35.28	32.55	27.34	33.63	31.29	32.68	30.47	20.49	23.98	25.64	31.21
B08	1.51	38.69	30.57	34.21	37.62	36.58	36.94	27.22	34.42	33.31	34.21	40.39	32.1	25.73	25.82	33.18
B09	1.51	31.1	32.1	36.1	33.1	28.1	34.1	25.1	35.1	39.1	37.1	29.1	27.1	38.1	30.1	26.1
B10	1.33	38.96	28.6	12.31	37.17	30.08	38.5	24.57	38.1	33.94	35.31	34.34	30.35	33.27	25.73	32.55
B11	0.65	37.48	31.56	32.37	35.55	35.28	35.55	27.61	35.55	33.2	32.81	39.58	29.64	23.93	23.44	34.21
B12	1.34	16.01	20.98	27.75	20.98	19.59	24.12	25.2	32.73	32.05	32.15	38.25	29.32	23.63	23.63	31.92
B13	0.62	31.1	32.1	36.1	33.1	28.1	34.1	25.1	35.1	39.1	37.1	29.1	27.1	38.1	30.1	26.1
B14	1.37	39.65	24.44	35.42	36.94	37.48	40.87	28.97	35.7	33.42	36.25	37.48	32.69	25.69	23.45	0.00
B15	0.27	34.97	39.84	15.72	36.9	34.91	0.00	28.92	31.7	36.18	33.94	26.13	0.00	27.17	25.55	30.03
B16	1.76	30.98	24.56	29.5	34.16	31.48	32.59	22.36	34.29	31.56	30.7	31.25	27.75	24.51	22.63	29.99
B17	0.88	34.53	26.75	32.82	35.74	35.06	34.8	27.21	35.37	32.1	33.13	37.52	28.92	25.42	24.21	31.87

B18	1.17	34.25	28.86	31.34	34.8	35.32	33.58	26.72	33.9	31.07	32.15	31.18	26.54	23	23.08	32.37
B19	0.59	36.04	30.58	35.51	36.36	35.85	38.87	29.54	36.98	34.93	34.29	41.73	34.12	22.51	23.7	35.15
B20	0.95	35.94	26.35	31.16	36.14	33.27	36.76	28.65	36.76	33	34.16	43.98	31.52	24.66	24.66	0.00
B21	1.71	34.34	24.38	32.42	34.48	33.27	36.58	27.96	35.38	34.35	33.45	32.24	13.32	24.35	23.22	0.00
B22	0.77	34.12	33.76	34.43	37.76	33.27	37.35	29.39	36.63	35.69	35.19	36.9	31.42	28.34	25.73	35.06
B23	1.68	33.31	23.94	32.4	36.53	35.62	35.49	28.43	36.62	32.53	35.54	43.86	28.68	24.86	23.72	20.23
B24	1.71	35.78	24.79	32.77	35.19	33.27	34.88	29.05	35.32	31.55	33.94	42.33	31.08	24.21	23.5	23.03
B25	0.88	34.97	30.17	31.56	35.98	33.27	35.11	25.77	35.11	31.59	33.71	35.96	29.98	25.06	25.5	34.87
B26	0.77	31.56	17.56	29.28	33.63	33.27	31.75	27.48	34.75	31.07	32.01	0.00	0.00	23.05	20.45	32.82
B27	0.83	36.09	26.65	30.93	36.07	33.27	35.42	26.58	36.63	34.55	34.21	33.81	26.36	26.45	22.85	33.27
B28	0.84	33.31	26.81	31.01	36.17	33.27	34.39	11.11	34.39	30.03	33.18	39.14	35.96	20.35	19.73	15.73
B29	0.72	33.85	29.14	31.8	35.81	33.27	35.67	26.9	35.04	34.61	33.44	30.48	21.73	24.84	23.89	32.69
B30	0.62	33.27	27.89	31.12	35.19	33.27	33.58	26.71	34.25	32.86	35.59	36.54	27.66	23.85	23.14	32.15
C01	1.01	33.49	28.9	30.66	34.03	34.55	34.43	25.8	33.94	34.48	34.02	12.65	0.00	23.29	22.19	0.00
C02	0.66	36.94	26.59	32.91	36.85	36.27	36.72	28.88	13.54	31.87	32.91	30.17	17.36	24.84	22.47	24.03
C03	1.01	36.14	26.59	33.36	36.09	35.73	37.29	28.16	33.07	35.82	35.33	39.49	30.35	24.48	22.33	0.00
C04	0.96	35.87	23.54	31.12	35.09	34.97	35.64	27.43	32.91	37.84	35.95	33.67	30.57	25.6	20.53	0.00
C05	0.81	37.02	28.86	33.09	37.7	34.39	38.87	29.8	34.7	33.13	34.16	37.21	33.22	25.02	22.01	35.05
C06	0.49	34.52	30.85	25.29	36.9	35.01	37.39	28.65	36.13	31.83	33.8	36.47	30.45	24.51	23.3	35.15
C07	0.77	35.33	29.45	30.43	35.91	33.67	36.13	29.01	32.91	30.8	32.17	40.8	30.76	24.21	21.16	31.52
C08	0.61	38.55	28.65	30.98	37.75	37.08	37.75	29.27	37.61	34.61	35.01	37.08	28.29	26.3	24.62	35.33
C09	0.59	37.16	28.74	31.47	35.77	33.8	36.68	29.73	33.91	32.1	34.55	42.44	32.63	24.61	22.94	36.53

C10	1.16	35.37	27.56	32.14	35.47	34.92	34.3	30.49	34.3	31.01	34.17	38.82	27.84	23.85	20.76	33.67
C11	0.78	35.19	28.2	31.88	36.36	35.94	37.68	29.77	31.88	32.82	32.98	26.94	20.11	24.61	22.73	25.87
C12	0.75	38.51	27.96	30.57	37.11	35.72	38.96	30	35.86	33.43	34.66	36.76	28.51	25.1	22.56	34.11
C13	1.39	34.41	27.93	32.14	36.63	36.72	36.63	28.64	34.15	32.5	33.79	29.22	10.17	24.57	22.24	19.1
C14	0.66	35.18	34.56	38.33	35.19	34.87	40.04	28.73	39.63	48.99	38.33	35.01	26.45	40.46	28.06	39.01
C15	0.98	34.88	30.18	31.91	34.97	33.53	35.96	27.84	35.55	33.94	35.55	39.23	30.66	23.94	22.33	35.78
C16	1.57	35.24	32.14	31.57	34.83	35.06	36.54	29.68	34.39	35.11	35.24	38.42	28.38	25.96	22.99	32.5
C17	0.33	34.84	30.67	32.95	36.41	35.73	36.54	27.29	33.54	35.06	36.36	40.75	29.5	24.97	23.22	31.86
C18	0.3	45.51	30.85	35.11	44.66	34.88	49.49	33.9	39.77	40.35	39.86	43.66	33.4	31.95	25.5	41.82
C19	0.94	34.61	40.82	35.22	36.9	36.85	42.77	27.49	37.39	33.71	37.53	36.13	27.53	29.91	25.01	37.89
C20	0	39.75	39.75	36.13	38.91	38.67	41.7	31.34	35.78	35.64	35.46	41.34	33.79	26.23	25.15	36.89
C21	0.55	38.42	31.16	34.79	37.7	39.28	37.17	23.61	34.88	33.67	34.79	42.95	32.86	24.12	23.62	36.93
C22	0.6	35.33	28.92	31.32	36.67	36.23	36.67	27.84	34.5	35.15	35.95	32.45	28.27	0.00	20.62	34.16
C23	0.46	36.13	33.94	33.13	37.8	37.26	36.72	28.73	34.16	35.42	34.38	35.4	29.91	23.99	22.18	33.49
C24	0.04	36.74	31.28	30.61	37.97	38.29	36.05	33.25	37.66	35.06	38.06	14.36	0.00	27.66	24.03	23.09
C25	0.39	33.4	33.18	33.27	37.93	35.69	39.98	28.07	34.48	32.87	33.8	34.25	27.13	24.26	21.39	35.24
C26	0.48	37.39	33.98	33.31	38.4	37.41	37.39	28.55	35.46	35.24	35.11	39.84	28.92	24.93	22.1	34.75
C27	0.39	40	37.31	38.69	39.63	36.87	41.6	30.93	35.87	39.22	40.39	44.15	34.7	27.88	23.84	36.68
C28	0.58	39.36	26.08	35.02	39.01	39.32	39.71	30.62	35.89	36.54	37.95	0.00	0.00	27.43	26.81	0.00
C29	0.83	37.43	32.49	34.6	37.88	37.21	36.81	30.54	36.81	34.28	36.85	42.23	30.26	25.06	23.24	34.39
C30	0.34	37.53	36.13	34.52	37.3	36.04	37.3	31.11	36.13	33.71	36.9	39.83	30.69	27.84	24.35	36.13
C31	0.45	36.13	32.91	33.22	38.33	36.17	39.39	31.87	35.38	34.03	36.14	39.12	28.36	29.05	24.62	32.6

D01	0.49	37.26	32.14	33.49	37.25	36.66	37.26	31.75	34.7	34.84	34.97	31.87	13.58	23.12	23	35.78
D02	0.94	38.32	35.09	34.7	36.27	36.45	38.42	31.61	34.97	34.88	34.7	34.75	29.95	25.01	23.76	35.51
D03	0.55	36.9	33.4	33.13	36.76	35.1	36.36	34.08	34.34	35.11	35.06	38.52	30.3	21.39	22.29	31.56
D04	0.81	32.48	31.52	32.48	35.11	35.87	34.75	29.9	34.97	34.06	33.98	40.79	19.09	23.85	21.07	37.79
D05	0.85	34.48	29.54	30.89	36.45	36.04	36.32	28.92	34.52	35.19	35.91	35.42	26.27	25.69	23.13	31.87
D06	1.18	37.53	34.57	32.35	37.66	36.67	39.59	30.84	36.67	36.23	35.11	31.7	29.64	26.14	24.62	26.94
D07	1.42	38.15	30.44	34.61	37.92	34.75	41.42	34.25	36.8	34.57	34.61	42.77	32.28	28.25	25.15	35.91
D08	1.07	34.29	24.52	33.09	35.74	35.46	35.1	24.03	33.48	36.13	36.63	0.00	0.00	26.64	23.36	0.00
D09	0.66	38.25	33.17	35.55	37.34	38.82	27.42	32.01	35.55	36.68	33.86	37.71	32.5	28.51	24.65	38.95
D10	1.06	34.57	30.42	31.29	36.54	37.3	35.73	28.24	34.43	34.07	35.18	38.33	27.75	24.43	24.08	34.25
D11	0.83	38.13	36.08	38.57	39.93	39.55	34.85	30.06	38.57	34.7	37.16	37.28	31.89	29.78	25.53	39.27
D12	0.79	38.14	33.67	33.96	40.07	40.39	39.27	32.65	36.69	36.26	36.31	33.82	12.36	27.63	27.56	35.37
D13	0.66	41.2	40.92	37.06	40.92	37.77	44.31	28.97	38.56	38.75	37.39	41.29	32.54	26.15	24.12	36.68
D14	0.98	40.07	30.9	36.88	40.12	38.05	41.01	31.51	38.05	38.24	36.88	40.21	32.78	26.9	22.85	37.58
D15	0.49	36.03	35.61	35.28	38.71	37.3	41.24	30.05	37.82	35.11	37.25	36.88	30.72	27.04	23.05	39.13
D16	1.22	39.55	28.84	34.61	37.9	39.32	40.73	31.27	35.18	38.42	35.75	31.61	31.05	21.59	21.86	29.91
E01	0.69	19.27	13.12	23	29.76	22.15	26.45	13.44	34.7	33.86	38.99	0.00	0.00	21.86	10.07	0.00
E02	0.6	38.15	31.33	33.86	39.43	40.43	41.48	33.86	42.65	40.92	39.4	42.11	33.02	30.98	26.9	34.83
E03	1.06	41.97	31.12	35.37	40.02	39.29	40.02	31.84	38.19	35.94	39.64	41.65	30.67	29.21	25.77	33.58
E04	1.14	37.58	27.6	34.92	39.69	37.99	38.15	32.26	39.45	40.26	39.37	43.74	33.06	31.13	27.13	25.71
E05	0.5	38.15	31.32	35.32	38.28	36.15	38.11	29.54	36.41	36.12	35.09	39.83	28.17	28.22	28.6	32.87
E06	0.77	36.72	27.47	33.68	40.69	37.55	35.37	30.51	34.19	39.54	36.83	43.97	29.25	31.65	28.88	0.00

E07	0.87	36.06	26.24	34.15	39.41	36.87	34.15	29.35	34.15	34.71	37.44	42.38	27.82	28.74	20.74	0.00
E09	1.1	40.92	31.32	34.98	39.93	39.65	38.94	29.73	34.98	32.02	34.98	45.57	31.7	27.52	26.52	35.79
E10	0.78	41.8	32.22	35.56	43.31	39.23	42.03	31.04	38.52	40.57	40.06	45.44	31.89	30.76	28.55	35.98
E11	1.99	35.82	28.92	33.72	40.07	37.09	36.83	32.41	33.72	32.69	34.84	45.33	36.12	29.91	29.1	37.66
E12	0.45	41.33	30.58	35.46	39.65	42.04	41.3	33.44	36.96	35.98	37.67	35.41	25.07	29.02	31.04	38.94
E13	0.92	26.95	18.34	35.7	39.82	31.98	21.99	30.75	39.51	34.01	35.7	47.39	31	31.31	27.65	34.86
E14	0.49	39.46	26.25	36.17	42.47	40.24	42.7	33.72	42.28	39.32	42.19	49.39	33.58	31.98	27.53	36.59
E15	0.71	40.77	27.7	35.45	42.01	39.3	41.91	34.9	39.97	33.88	37.39	39.3	20.96	28.92	27.04	17.26
E16	1.25	38.38	32.82	37.41	42	39.6	44.73	30.52	42.62	33.43	36.87	40.92	33.53	28.17	27.98	37.29
E17	0.56	40.68	38.1	36.16	41.39	38.17	42.6	35.32	38.52	42.46	37.05	46.56	37.48	31.7	28.45	39.55
E18	1.3	36.83	33.19	37.82	39.98	38.36	41.77	29.71	37.82	38.56	40.96	40.86	35.31	31.04	27.61	37.95
E19	0.56	40.4	26.07	38.05	42	39.43	43.63	35.14	39.43	36.4	38.05	39.43	19.9	30.8	29.26	0.00
E20	0.6	40.78	26.29	36.16	41.39	39.26	39.13	31.68	40.64	37.7	39.27	38.47	17.96	28.79	26.66	0.00
E21	0.92	43.93	0.00	39.42	43.93	39.3	42.27	34.7	40.44	36.77	39.42	45.44	30.85	28.17	30.33	37.25
E22	0.58	43.5	34.24	36.65	42.24	39.65	43.32	31.47	37.49	37.04	37.51	49.77	34.52	29.63	28.97	38.04
E23	0.58	38.93	32.81	37.62	40.54	41.02	41.02	30.06	39.6	40.4	37.62	45.57	32.31	30.1	29.35	36.45
E24	0.54	38.71	28.5	34.34	38.67	38.23	37	29.91	35.04	34.1	34.61	40.02	30.34	29.35	28.4	34.2
E25	1.11	39.04	32.03	34.67	40.26	41.06	40.45	31	37.28	33.47	37.77	41.06	32.78	30.06	28.13	35.65
E26	0.99	41.47	30.66	37.25	39.51	41.48	40.77	32.22	39.15	36.02	37.39	42.09	31.99	28.78	27.09	37
E27	0.83	38.05	29.4	35.7	39.27	39.51	38.33	29.07	36.68	36.03	39.08	31.37	19.74	0.00	28.55	0.00
E28	0.49	38.61	31.42	35.88	38.61	38.98	40.82	28.12	37.02	37	37.82	40.59	30.18	29.81	27.47	35.46
F01	1.6	44.87	32.21	37.43	41.73	39.55	44.59	35.16	43.08	38.08	39.55	52.04	36.26	32.92	32.36	37.34

F02	0.69	42.66	30.57	38.33	42.64	41.58	42.74	32.45	41.46	37.38	40.87	46.35	34.24	31.38	29.3	36.78
F03	1.7	42.99	34.48	37.99	42.8	39.09	47.34	36.59	43.79	38.85	46.47	50.61	35.84	31.41	30.43	38.94
F04	0.61	38.94	29.44	37.1	43.65	41.64	42.99	33.44	41.95	38.11	41.3	35.84	13.49	30.56	28.36	36.82
F05	0.41	42.89	32.01	37.82	41.75	39.45	43.27	33.72	41.06	37.53	40.83	44.2	33.39	31.27	29.26	39.37
F06	0.34	43.69	37.27	40.16	42.33	41.67	45.01	37.25	44.02	39.65	40.16	41.67	29.01	35.28	32.66	0.00
F07	0.57	39.51	31.8	35.61	40.02	38.66	43.36	30.71	40.02	37.25	40.87	46.47	33.89	30.85	28.26	35.31
F08	0.94	39.93	28.51	36.54	39.74	40.92	43.35	33.53	41.81	40.58	40.73	41.9	29.58	30.65	28.62	34.24
F09	1.2	38.46	31.44	34.88	36.4	39.11	37.23	30.95	35.36	32.26	32.43	40.99	33.14	26.29	26.55	35.58
F10	1.14	42.38	27.23	42	42.8	40.06	44.59	32.36	42.56	38.67	40.62	44.87	36.31	31.74	30.01	38.09
F11	1.3	40.87	34.71	35.28	41.47	39.74	41.84	34.5	41.84	37.72	40.02	46.19	32.87	30.29	27.57	32.87
F12	0.76	40.31	30.51	36.73	39.55	39.27	43.55	31.14	39.08	38.94	42.38	46.71	34.2	29.63	28.17	35.84
F13	1.61	45.28	37.44	39.6	40.68	40.85	43.79	32.5	40.96	35.32	41.53	37.29	12.98	29.44	28.08	36.02
F14	1.06	41.67	33.25	37.47	41.06	39.8	42.47	33.8	42.75	36.96	41.1	43.03	32.83	30.05	29.54	37.72
F15	1.09	41.68	38.89	38.94	42.28	40.31	44.59	32.12	44.49	38.24	41.1	46.99	35.65	30.43	27.37	35.65
F16	1.25	38	25.44	35.72	38.33	39.66	42.38	31.32	39.01	37.44	42.94	35.56	24.69	27.8	25.31	28.84
F17	0.8	36.12	29.21	35.84	37.53	38.24	37.53	31.51	38.05	34.01	38.89	43.02	28.26	30.71	28.03	33.77
F18	0.36	37.33	35.03	37.69	38.93	38.23	39.83	31.88	39.7	36.16	40.82	38.23	31.56	31.37	26.95	37.96
F19	0.28	40.73	37.01	39.97	41.42	39.13	44.78	33.99	40.57	37.91	40.36	49.04	37.48	30.9	29.3	38.33
F20	0.79	40.53	32.16	39.08	39.27	39.21	41.35	31.24	40.21	35.32	39.08	45.35	33.15	29.11	27.28	35.89
F21	1.37	38.09	26.53	36.03	39.6	40.3	39.51	32.53	39.51	36.68	38.57	42.23	31.23	26.99	26.95	35.69
F22	1.13	41.67	32.74	35.79	41.57	39.42	45.43	31.98	42.52	40.29	41.68	32.21	11.67	29.14	27.65	36.17
F23	1.44	37.25	29.41	35.22	38.23	39.55	40.31	32.07	39.88	36.83	38.15	37.86	22.38	29.25	29.35	35.89

F24	1.46	39.6	28.22	35.73	40.62	38.85	41.43	32.9	41.3	36.78	40.07	42.89	31.88	30.76	30.1	36.63
F25	1.51	38.32	26.43	35.55	40.19	41.34	40.53	28.32	40.16	38.21	41.44	0.00	0.00	29.24	28.07	0.00
F26	0.71	36.55	22.19	34.32	36.39	37.2	44.06	31.09	37.25	36.03	40.16	37.96	37.48	27.09	24.6	32.96
G01	0.59	36.69	32.84	34.24	37.51	35.85	38.58	29.78	37.01	33.75	36.11	43.58	30.77	25.93	25.56	32.26
G02	0.89	37.09	27.88	34.08	37.39	36.6	37.39	28.82	37.6	33.34	35.56	36.23	28.58	24.81	22.66	32.05
G03	0.29	35.11	28.7	32.92	37.01	37.39	37.01	28.36	36.93	33.96	36.44	38.13	29.45	24.24	22.42	32.3
G04	0.97	35.74	29.57	31.89	35.73	36.11	35.73	28.07	37.34	33.54	34.31	35.98	28.83	23	21.38	33.21
G05	0.44	34.33	29.53	31.93	36.19	35.03	35.44	25.96	38.33	34.32	36.85	37.01	30.89	24.65	21.13	32.01
G06	0.72	37.97	32.59	32.38	37.72	36.55	38.22	31.23	35.74	33.58	37.22	35.31	30.65	24.24	22.75	0.00
G07	0.76	35.89	27.41	32.25	36.03	35.44	36.8	27.67	38.09	34.66	37.13	37.56	27.04	25.39	22.75	31.81
G08	0.63	35.61	27.46	32.74	36.43	36	36.73	28.46	36.19	31.93	35.49	37.64	27.74	24.24	22.75	29.03
G09	0.74	36.03	27.91	32.67	36.77	34.91	36.77	28.71	35.36	32.18	35.16	35.57	30.02	25.06	23.37	31.85
G10	0.68	36.56	29.04	35.28	35.98	36.81	35.98	28.37	36.52	33.15	37.36	38.96	31.09	24.16	23.45	33.38
G11	1.4	37.14	28.63	32.26	34.23	30.81	36.15	29.69	37.29	32.9	37.1	42.23	30.65	25.79	22.58	0.00
G12	0.96	34.62	29.32	32.05	35.61	34.94	35.61	28.04	35.61	33.08	36.31	41.3	28.04	23.04	20.85	28.04
G13	0.86	37.43	31.01	33.17	37.3	37.17	37.8	30.76	36.81	35.16	34.91	41.36	31.51	24.6	20.8	30.76
G14	0.6	35.24	29.23	32.87	35.78	35.56	37.39	28.75	36.14	32.96	36.1	35.56	27.95	25.23	22.25	28.75
G15	1.54	34.66	27.51	32.05	36.19	34	36.19	27.17	36.19	34.33	35.52	35.18	27.79	23.62	21.96	31.84
G16	1.15	33.62	30.29	31.3	34.99	35.48	37.75	28.71	37.02	35.16	36.01	37.44	27.29	27.29	23.73	33.01
G17	0.52	30.14	28.45	29.45	30.98	32.38	34.66	23.2	34.66	35.88	34.24	30.15	25.73	24.48	22	30.48
G18	0.68	33.25	28.25	29.62	34.62	35.35	35.81	29.69	36.96	35.07	36.51	37.71	26.47	26.93	25.06	31.09
G19	0.65	34.62	29.78	31.91	35.57	34.65	36.15	28.63	35.98	30.32	33.54	33.21	28.08	24.73	22.58	32.14

G20	0.26	37.18	26.31	31.14	36.41	36.52	37.51	31.16	38.17	32.58	35.62	30.93	17.86	28.6	25.89	35.23
G21	0.84	38.54	32.79	35.69	39.44	39.96	38.3	32.5	37.35	33.49	35.07	28.37	11.17	27.46	26.98	37.26
G22	0.91	37.11	30.1	33.6	38.49	37.35	38.12	30.36	34.83	34.16	33.6	36.97	28.47	26.51	27.37	30.89
G23	0.78	33.5	30.93	32.63	34.4	34.98	34.4	29.27	34.04	30.85	33.46	33.62	26.58	24.57	22.75	31.93
G24	0.86	20.72	26.84	32.5	31.98	23.62	30.54	29.7	38.01	34.36	34.84	0.8	0.62	26.03	25.76	35.49
G25	0.86	33.12	30.29	31.87	34.94	34.86	34.94	29.42	34.94	36.11	37.71	34.86	29.2	24.37	23.98	35.99
G26	0.7	30.65	29.53	30.19	32.47	32.88	33.82	25.96	33.82	33.98	34.7	32.09	24.99	24.57	23.33	30.9
G27	1.39	30.15	29.6	28.79	29.6	31.1	33.17	26.38	33.17	32.46	32.71	31.1	25.51	23.86	22.29	29.45
G28	0.79	28	24.24	29.41	29.2	29.65	30.73	21.01	32.91	31.39	32.31	31.02	23.48	23.32	21.09	30.18
G29	0.66	14.85	23.74	27.62	23.74	18.7	25.6	23.59	31.26	29.69	32.05	0.00	0.00	23.78	13.24	0.00
G30	0.91	33.13	28.74	31.06	34.04	34.12	34.98	29.86	34.98	34.4	36.47	33.7	28.04	24.07	22.46	31.8
G31	0.67	33.13	24.71	31.06	34.74	26.39	34.74	29.98	34.74	31.72	34.49	33.33	27.79	24.32	23.33	0.00
G32	0.6	30.85	23.13	30.15	32.26	33	32.26	27.59	33.87	31.55	34.66	13.48	10.75	24.77	23.16	0.00
G33	0.74	31.81	23.37	30.03	33.62	34.04	33.62	26.54	33.62	30.15	33.79	0.00	0.00	23.66	21.92	0.00
H01	0.62	35.47	35.47	32.12	36.71	36.81	35.42	30.55	38.5	36.84	38.95	34.75	28.19	29.76	26.59	33.73
H02	0.86	33.55	29.44	32.06	37.83	36.45	36.45	29.78	38.4	35.82	37.76	39.54	29.35	27.52	25.25	30.00
H03	1.52	40.27	26.47	34.31	40.08	38.83	45.68	38.03	38.43	34.13	41.64	43.67	39.9	31.44	28.04	0.00
H04	1.31	36.18	31.14	33.99	38.01	38.5	40.28	30.07	40.69	40.28	42.37	39.64	27.08	31.18	25.51	33.59
H05	0.47	34.53	27.48	32.57	36.58	37.25	37.7	27.57	37.92	34.98	36.36	35.78	27.48	0.00	22.49	31.32
H06	0.84	38.19	31.67	35.19	33.94	40.28	39.57	28.06	39.57	36.71	38.9	38.4	9.95	26.59	25.65	35.1
H07	1.02	36.58	31.05	33.32	39.02	39.87	38.41	30.59	38.95	34.19	37.56	25.32	24.32	21.63	19.18	28.86
H08	1.4	36.89	25.75	35.15	37.39	39.3	40.42	31.65	40.24	35.87	39.13	10.07	0.00	28.77	16.55	0.00

H09	0.94	34.31	28.77	32.65	35.16	35.47	37.25	32.74	37.34	35.06	36.09	27.79	0.00	28.11	26.41	25.12
H10	0.79	33.1	30.11	32.83	35.68	36.4	35.68	31.53	35.68	31.76	34	34.74	28.55	26.41	26.89	30.91
H11	1.02	34.26	30.1	33.19	36.45	36.67	36.45	29.18	36.45	35.19	35.87	34.84	27.04	24.94	22.13	30.56
H12	0.89	33.33	28.41	30.88	34.4	35.16	34.52	28.64	35.2	32.26	34.98	32.65	25.21	24.66	23.91	31.32
H13	0.85	33.1	20.24	29.57	33.95	34.15	33.95	26.06	35.29	31.44	34.16	32.12	25.47	24.66	22.39	33.2
H14	1.28	33.68	28.15	32.43	34.75	33	34.75	24.18	35.37	32.34	34.93	29.67	25.25	24.05	23.32	29.27
H15	0.77	36.26	27.83	34.21	36.23	35.91	36.94	33.22	35.73	32.47	34.21	34.89	31.9	25.73	24.54	33.22
H16	1.22	37.56	33.85	34.64	38.46	37.65	39.63	30.2	40.69	37.18	38.4	40.44	31.18	27.12	24.62	32.61
H17	0.64	32.92	29.4	31.18	34.89	34.17	34.21	26.01	35.91	31.5	34.58	33.23	25.56	24.67	22.48	33.06
H18	0.89	35.34	32.2	33.52	38.3	35.96	36.94	27.12	37.5	34.78	37.27	27.42	14.73	26.63	25.41	33.56
H19	1.01	37.74	28.27	37.41	40.46	39.33	40.46	32.21	40.46	35.72	39.56	37.88	30.84	26.34	23.72	0.00
H20	0.79	33.75	27.8	33.93	35.95	35.77	37.17	25.92	38.02	35.53	36.66	35.77	30.28	26.86	25.03	0.00
H21	0.55	31.76	31.76	33.46	38.07	35.83	41.29	31.17	38.7	31.36	36.41	40.31	29.65	28.35	26.64	33.5
H22	0.61	36.05	31.16	34.64	37.83	37.6	37.83	29.75	37.83	35.34	36.89	37.6	29.63	25.17	23.2	32.11
H23	0.95	30.79	35.39	35.53	40.13	32.44	40.13	31.17	41.67	37.54	39.68	38.81	31.4	27.29	27.23	38.53
H24	0.98	37.74	35.67	37.45	37.4	38.62	38.25	29.86	40.34	37.78	39.52	35.86	31.45	25.92	22.78	34.83
H25	0.51	33.94	31.93	34.92	36.84	37.26	36.84	28.78	38.11	36	36.07	36.33	31.22	25.02	22.31	34.5
H26	1.03	35.66	32.67	33.38	38.03	38.57	40.07	32.21	39.94	40.12	40.22	42	33.09	26.2	23.52	37.22
H27	0.54	33.7	29.81	32.14	36	35.67	32.95	28.08	38.62	36.19	37.82	35.06	29.95	24.89	22.27	34.78
I02	1.24	34.55	27.28	35.14	37.88	37.53	37.11	31.97	37.4	31.97	35.76	0.00	0.00	26.49	25.59	27.42
I03	0.76	36.85	32.67	35.9	41.39	40.27	41.39	31.97	38.39	35.56	36.46	41.62	32.58	29.44	29.06	38.11
I04	1.79	38.63	33.37	37.31	39.61	40.68	41.62	31.49	38.81	35.43	37.69	45.84	33.46	30.13	27.19	35.77

I05	1.31	40.18	37.48	35.81	38.98	39.09	38.86	31.31	37.26	36.45	35.81	39.09	34.08	29.91	30.8	39.04
I06	1.12	35.53	29.43	33.09	34.08	35.95	33.98	28.87	33.98	31.68	33.09	38.67	29.76	26.11	24.8	29.85
I07	1.11	41.2	26.76	35.4	40.6	40.16	41.48	31.68	38.11	36.33	37.87	39.09	30.61	21.84	28.27	0.00
I08	0.64	33.84	26.39	33.46	35.78	38.9	37.41	31.36	39.57	35.4	40.27	47.57	32.48	29.3	28.22	0.00
I09	1.69	39.37	32.81	35.19	38.86	40.78	40.27	29.8	36.14	34.49	35.62	43.35	34.12	28.78	31.08	35.72
I10	1.64	37.26	31.03	35.34	39.14	39.09	39.66	30.18	40.27	35.05	38.48	43.08	31.78	30.56	27.19	32.44
I11	1.36	33.05	30.13	34.22	35.81	36.14	37.68	30.14	37.68	34.03	36.28	36.57	28.22	27.19	23.75	33.18
I12	2.22	37.13	32.21	35.95	36.28	38.43	38.67	32.95	38.58	33.19	37.69	45.98	34.59	28.83	26.39	34.02
I13	1.52	34.97	27.56	33.61	36.09	35.9	36.05	30.13	37.69	33.38	34.96	31.78	28.97	28.12	24.04	33.56
I14	1.38	33.12	28.41	34.08	36.89	37.91	36.89	32.21	38.11	32.33	36.74	34.97	30.23	29.06	25.69	34.12
I15	0.42	35.91	24.14	30.84	37.78	37.03	36.99	30.84	37.69	34.5	38.72	30.27	11.63	25.07	23.95	22.73
I16	1.47	34.92	30.37	31.6	37.87	37.29	37.87	29.72	39.56	33.93	38.86	34.17	11.26	27.94	24.7	13.09
I17	1.92	18.29	31.65	0.00	11.07	11.11	0.00	28.04	24.38	37.88	28.37	34.83	23.04	26.99	27.09	36.3
I18	0.52	34.12	29.3	31.49	36.52	35.17	35.2	29.67	36.51	32.01	36	39.23	29.06	27.01	25.41	33.56
I19	0	36.38	26.77	34.22	35.77	37.07	36.66	33	38.2	33.98	38.34	14.21	0.00	31.78	12.89	0.00
I20	0.83	37.31	29.81	34.88	38.11	37.31	38.11	30.84	37.36	32.06	35.72	39.66	28.97	29.58	26.44	31.92
I21	0.85	35.34	29.51	33.42	35.38	37.64	31.45	29.53	36.51	32.38	36.93	39.14	28.41	29.07	26.53	33.85
I22	1.02	36.19	32.16	34.72	38.53	39.04	40.64	28.21	39.52	38.61	39.94	37.97	33.98	31.5	27.14	35.3
I23	1.55	35.34	31.41	36	37.36	37.53	39.98	29.3	39.42	35.64	39	38.34	32.57	28.22	23.38	32.57
I24	0.93	36.09	31.07	35.11	38.53	39.75	38.53	33.42	38.53	33.33	35.85	44.3	31.26	30.24	29.44	33.42
I25	2.48	36.98	33.28	35.16	38.14	39.37	40.3	30.68	38.58	33.75	38.25	40.68	32.34	30.65	28.04	32.06
I26	1.24	36.05	30.35	35.25	39.04	39.28	38.48	32.53	38.48	34.58	38.81	48.98	30.61	30.41	28.17	33.73

I27	0.94	34.22	28.73	32.53	36.55	36.93	39.12	31.51	38.85	32.47	36.89	36.93	30.61	26.49	23.86	32.2
J01	0.87	32.27	30.05	29.55	33.29	33.31	33.9	28.51	36.75	33.71	36.66	33.31	24.71	24.83	21.58	28.6
J02	0.81	35.39	29.45	32.85	34.66	34.44	36.02	27.06	36.61	33.71	35.44	38.55	31.59	27.92	24.75	31.59
J03	1.17	38.01	29.92	34.29	36.87	38.36	40.77	30.29	36.66	31.81	35.8	37.74	31.04	28.83	23.44	0.00
J04	0.9	34.44	29.68	34.21	37.52	37.06	38.06	30.05	37.79	35.71	35.57	34.66	30.19	24.75	21.22	32.54
J05	1.24	36.42	31.5	31.95	38.83	36.58	38.83	32.24	38.47	32.94	38.05	34.89	22.48	28.24	25.03	0.00
J06	0.75	30.95	23.08	32.68	36.52	34.39	36.52	21.49	36.02	31.21	34.67	37.41	26.92	26.97	25.38	34.35
J07	1.26	31.45	28	33.89	33.13	35.47	34.66	29.41	35.21	31.23	35.56	33.17	14.13	27.42	24.12	31.27
J08	1.26	34.85	29.42	32.76	36.66	36.16	36.66	24.67	34.12	32.31	34.79	36.83	32.25	27.2	24.33	34.66
J09	0.9	36.25	29.05	32.99	36.52	37.42	37.42	27.09	36.16	34.44	35.12	34.66	31.27	27.29	24.89	33.67
J10	0.99	36.7	34.89	32.65	34.89	37.87	39.33	31.63	38.28	33.52	35.43	37.88	32.54	29.69	25.17	33.17
J11	0.89	11.41	25.29	24.08	19.73	12.7	21.81	29.87	34.21	30.35	34.24	0.00	0.00	27.24	24.53	0.00
J12	1.43	34.34	28.78	33.3	36.78	35.75	38.6	30.59	37.2	32.66	35.02	36.83	28.51	0.00	24.16	31.14
J13	0.24	35.98	30.99	33.31	37.56	38.19	38.42	27.7	39.1	34.66	36.2	36.65	28.18	29.41	27.01	32.13
J14	0.75	33.53	28.15	33.04	35.93	34.43	36.39	27.61	36.39	32.85	34.12	36.47	28.37	25.93	23.8	30.09
J15	1.34	34.66	29.12	32.94	35.74	35.66	35.74	28.74	35.3	32.95	35.56	37.34	28.38	26.1	21	29.64
J16	0.85	34.21	30.32	32.82	36.93	34.89	36.2	28.31	38.24	34.65	37.29	14.98	0.00	27.65	23.72	24.84
J17	1.16	32.09	29.69	32.62	34.57	35.66	34.61	30.27	36.15	33.12	35.56	36.12	30.77	26.65	23.8	33.53
J18	0.63	34.71	26.2	33.83	38.87	36.11	38.87	21.04	38.37	30.77	36.47	22.99	9.69	29.05	23.53	31.49
J19	0.04	38.82	34.03	35.89	39.64	40.27	37.28	31.41	36.52	35.16	35.89	38.92	32.9	23.56	25.16	36.25
J20	1.6	38.01	31.09	34.8	38.46	38.71	38.92	30.77	37.1	32.63	35.56	42.35	31.22	28.78	26.56	32.9
J21	0	36.56	31.48	34.53	37.2	38.19	36.66	29.44	35.93	36.76	38.05	37.65	31.63	23.84	26.52	33.98

J22	0	36.93	23.8	34.65	38.28	39.23	38.28	32.07	39.6	37.37	40.18	40.95	32.54	29.96	25.74	38.32
J23	0	34.57	29.78	34.26	38.78	38.1	36.84	28.51	35.03	35.75	36.47	38.1	30.9	25.6	26.61	32.99
J24	0	39.19	32.86	36.47	39.51	37.05	38.56	30.11	37.24	37.73	38.92	41.09	32.4	28.15	27.87	35.39
J25	1.13	36.38	27.92	32.4	34.41	35.93	35.88	29.07	35.88	30.68	34.27	43.61	29.96	25.57	24.38	32.54
J26	0.67	37.02	29.91	34.49	37.97	30.64	37.52	29.91	35.43	33.03	34.49	37.28	34.66	27.15	25.75	34.66
K01	1.06	34.84	29.28	31.99	35.52	36.83	35.25	24.38	35.3	33.78	34.35	42.04	29.82	25.93	24.21	31.59
K02	0.81	34.28	31.16	32.35	35.82	36.78	35.82	26.91	34.98	33.18	33.88	36.78	28.75	24.95	25.03	31.96
K03	0.7	35.77	26.17	32.6	36.96	35.1	34.72	26.38	34.72	33.53	34.28	38.92	29.94	26.04	24.28	32.18
K04	0.69	36.69	28.53	30.99	35.59	35.84	35.59	26.57	35.59	31.12	33.05	41.81	29.19	26.17	22.09	32.74
K05	0.49	36.69	31.46	35.98	38.61	34.8	37.96	28.89	35.98	31.43	33	37.83	30.34	26.83	24.15	34.41
K06	0.58	35.81	33.14	33.13	36.56	36.69	37.47	29.46	34.06	33.41	35.25	36.69	27.88	24.72	24.2	31.74
K07	1.02	33.4	26.04	31.42	34.84	32.47	32.47	26.43	34.18	30.82	31.96	36.29	28.27	22.75	21.31	32.62
K08	1.07	35.77	27.19	33.53	36.29	35.64	33.27	26.57	34.44	32.09	33.66	34.98	27	22.49	21.7	33.27
K09	0.43	34.72	22.22	31.68	36.69	34.99	33.53	27.14	33.53	32.22	33.26	38.39	27.22	25.25	23.94	34.06
K10	0.78	35.24	22.49	31.03	34.72	35.46	35.37	29.72	35.37	31.16	33.4	32.18	19.72	24.58	24.72	0.00
K11	1.27	36.66	30.38	34.5	37.04	38	39.71	29.59	31.96	35.59	35.64	34.27	22.97	26.48	25.25	0.00
K12	1.02	37.09	24.72	32.48	37.26	34.94	37.26	29.19	35.29	32.53	33.36	37.17	28.32	23.7	25.91	0.00
K13	0.67	34.85	19.99	32.39	36.6	35.55	35.72	26.95	36.11	32.74	34.28	38.98	27.57	25.86	25.07	0.00
K14	0.9	36.54	31.07	33.72	38.14	38.38	36.4	28.03	36.83	35.65	36.5	40.39	28.55	26.95	24.69	33.11
K15	0.66	39.09	28.64	37.24	38.93	39.98	38.93	29.44	38.65	36.68	37.24	41.57	30.57	26.25	26.05	17.02
K16	0.5	39.83	36.38	36.25	40.9	41.81	42	30.19	40.07	32.59	38.8	40.11	30.7	29.01	26.24	37.67
K17	0.57	37.99	27.54	35.23	38.8	36.92	39.22	28.45	37.86	33.7	36.45	40.31	30.52	25.91	24.36	15.52

K18	0.77	37.61	34.33	34.42	39.22	38.1	38.89	29.24	36.63	34.14	35.27	40.58	31.07	27.14	25.82	35.88
K19	1.77	35.91	30.26	32.4	35.68	36.73	35.68	25.24	35.41	33.3	33.07	35.86	30.51	25.75	23.24	34.99
K20	0.86	36.64	30.74	32.4	35.95	35.68	36	25.15	36	33.95	34.72	37.78	28.16	24.87	24.88	32.8
K21	2.32	34.81	27.83	33.9	36.36	35.64	37.32	26.65	36.64	35	35.55	35.49	29.53	25.55	24.42	34.18
K22	1.51	36.5	26.24	32.11	39.23	37.77	39.23	29.75	39.1	36.5	36.63	31.26	10.93	25.55	25.66	0.00
K23	0.55	36.77	28.89	32.79	38.82	37.63	37.46	29.8	37.46	35.4	35.41	30.62	11.07	26.25	25.01	0.00
K24	0.87	37.36	33.39	33.49	38.87	39.51	38.87	28.43	38.87	35.95	36.96	31.67	13.25	27.79	27.2	28.43
K25	0.55	35.81	36.59	32.54	40.19	38.41	38.77	29.39	38.32	35.66	37.59	36.54	32.67	24.74	25.97	33.76
K26	0.63	36.95	29.89	32.4	37.59	37.22	35	27.88	35.68	33.62	36.09	38	28.11	26.79	24.6	32.67
K27	0.75	33.62	30.88	31.16	37.18	35.8	35.13	25.83	36.36	34.76	34.63	40.14	29.94	26.57	26	32.26
K28	0.7	37.03	19.96	33.49	38.42	38.54	36.5	27.75	37.59	35.68	36.23	0.00	0.00	26.38	25.16	32.95
K29	0.39	33.08	13.79	34.17	39.78	37.03	38.27	0.00	37.99	31.44	34.17	18.98	29.42	26.66	26.93	32.07
K30	1.2	18.86	27.93	28.3	28.98	18.71	26.66	28.62	35.13	35.66	36.91	35.41	12.85	27.47	24.47	0.00
L01	0.97	31.7	28.13	29.04	35.44	31.69	30.87	27.58	32.33	31.42	32.1	34.75	28.77	24.73	36.85	30.6
L02	1.25	15.75	13.02	17.12	29.32	21.83	17.8	11.79	27.26	28.22	29.73	31.24	24.84	19.45	19.87	28.54
L03	0.44	36.08	30.82	33.11	36.08	36.17	36.17	28.04	36.17	34.53	33.11	33.79	29.09	23.64	23.1	34.61
L04	0.83	25.81	28.13	28.95	31.6	29.82	31.6	23.52	31.6	27.72	29.68	29.82	25.71	21.23	19.66	31.47
L05	0.77	30	22.32	29.32	31.92	31.69	30.69	27.04	33.02	30.83	32.19	40.6	30.64	21.63	20.13	24.47
L06	0.68	34.53	29.3	32.51	35.07	36.62	34.43	28.64	34.43	32.74	32.51	33.43	28.86	21.65	19.73	30.41
L07	1	31.74	24.25	29.55	33.62	31.69	32.38	27.22	33.06	32.15	31.42	30.19	28.87	20	18.09	0.00
L08	1.16	31.79	29.14	30.14	32.11	32.88	31.79	26.49	31.05	31.78	30.14	30.69	26.07	19.13	18.9	28.68
L09	0.75	28.81	22.29	28.64	31.51	32.61	30.83	24.38	28.64	30.55	30	0.00	0.00	19.16	19.04	0.00

L10	1.08	29.82	22.47	29	33.2	31.74	33.2	11.07	32.7	29.32	32.06	40.92	21.78	23.47	19.71	33.98
L11	0.92	33.52	30.37	30.36	35.25	36.31	33.38	26.9	33.43	33.75	33.29	33.29	30.14	22.78	20.86	32.1
L12	0.47	32.06	24.16	31.64	36.53	33.1	32.83	12.7	32.83	30.09	32.06	34.34	29.3	22.92	20.05	32.46
L13	0.83	33.88	27.35	30.83	33.98	33.46	33.98	26.44	33.33	31.12	23.53	34.71	29.05	20.41	18.9	31.27
L14	0.69	32.83	30.05	32.01	34.16	34.29	32.46	25.61	32.01	30.62	31.33	39.36	28.9	19.92	19.41	29.69
L15	1.79	13.43	20.87	23.43	19.23	14.84	19.45	23.57	29.31	28.86	20.34	22.2	26.72	20.08	19.59	0.00
L16	1.15	34.8	30.96	33.34	36.49	33.56	34.43	28.94	34.43	32.6	32.88	33.47	29.14	21.7	20.36	29.78
L17	0.83	29.68	23.96	27.94	31.72	30.14	28.08	15.93	32.38	29.18	31.34	30.14	24.62	21.37	20.56	23.93
L18	1.22	32.63	24.74	29.59	32.01	32.15	31.88	25.52	34.29	32.79	31.88	32.42	27.63	19.32	20.24	23.52
L19	0.65	10.82	27.51	27.76	19.91	12.91	20.82	28.17	31.28	34.71	36.26	9.73	0.00	27.86	27.22	25.44
L20	0.9	30.78	27.31	28.95	34.29	31.6	31.33	25.48	31.33	30.91	30.5	30.68	26.45	19.98	21.87	31.24
L21	1.4	35.35	28.63	31.28	33.84	35.07	34.8	28.64	34.29	34.26	33.34	35.07	28.64	25.21	22.17	0.00
L22	1.08	34.98	33.47	32.29	36.63	35.94	36.63	27.9	36.63	35.76	36.9	32.01	30.1	22.24	21.19	30.59
L23	1.23	35.34	28.57	31.47	35.39	35.53	33.33	26.21	34.29	32.1	31.47	32.57	27.63	21.19	20.4	31.19
L24	1.24	34.76	30.82	30.95	36.58	35.8	32.76	29.95	33.41	31.51	31.79	32.32	28.77	21.51	20.4	34.94
L25	1.1	20.1	30.35	34.07	23.29	24.7	28.68	31	33.34	36.57	37.03	14.02	0.00	22.33	15.21	28.13
L26	1.63	32.18	29.99	31.92	34.25	35.44	34.25	27.91	34.25	33.43	31.92	31.15	28.48	21.23	19.84	31.24
L27	1.5	33.56	28.5	31.51	35.94	34.53	33.98	26.72	33.84	33.15	32.88	34.53	28.91	21.09	21.37	32.06
L28	0.95	20.78	27.72	30.55	25.81	24.93	27.17	27.81	35.81	34.8	34.25	12.19	0.00	20.14	14.66	27.13
L29	1.4	31.6	24.89	29.45	33.7	34	32.32	24.11	32.32	28.77	30.42	28.5	22.87	20.28	17.54	25.94
L30	1.27	30.14	33.5	32.47	38.77	38.48	38.77	27.13	36.58	33.83	33.38	32.45	27.95	24.1	17.53	38.64

* RpoS data is from Valencia et al. [1]

Table S2. Bacterial survival to dehydration and acid stress. Isolates with RpoS levels > 1 SD were submitted to acid or dehydration stresses. Values represent the percentage of bacterial survival.

Strain	RpoS	Acid	Dehydration
A05	1.72	81.56	62.61
A11	1.34	87.41	
A19	0.50	88.31	3.47
A22	1.34	85.51	16.41
B06	2.21	0.00	5.25
B07	1.62	79.52	44.65
B08	1.51	85.52	39.09
B09	1.51	66.86	13.86
B12	1.34	99.31	24.98
B14	1.37	73.33	17.15
B15	0.27	59.50	65.14
B16	1.76	80.16	19.53
B21	1.71	77.34	38.10
B23	1.68	87.52	43.90
B24	1.71	70.63	12.57
C06	0.49	76.04	25.13
C13	1.39	67.51	
C16	1.57	82.00	47.51
C17	0.33	0.00	1.40
C18	0.30	0.00	0.54
C20	0.00	5.97	0.63
C23	0.46	77.10	34.54
C24	0.04	0.00	0.04
C25	0.39	1.40	1.41
C26	0.48	79.97	
C27	0.39	83.68	
C30	0.34	84.75	41.84
C31	0.45	89.67	
D01	0.49	77.18	
D07	1.42	73.35	4.55
D15	0.49	94.34	
E08	0.42	55.03	
E11	1.99	79.61	49.00
E12	0.45	78.21	
E14	0.49	141.59	
E28	0.49	112.69	
F01	1.60	93.25	
F03	1.70	53.02	59.33
F05	0.41	72.26	

F06	0.34	8.74	11.42
F13	1.61	60.40	
F18	0.36	14.99	
F19	0.28	0.00	35.47
F21	1.37	96.34	
F23	1.44	0.00	62.24
F24	1.46	88.82	39.33
F25	1.51	72.17	
G03	0.29	70.97	5.13
G05	0.44	76.38	
G11	1.40	49.86	
G15	1.54	7.93	65.92
G20	0.26	0.00	34.19
G27	1.39	5.28	96.16
H03	1.52		77.35
H08	1.40	94.11	
I04	1.79	77.22	40.39
I09	1.69	15.72	1.61
I10	1.64		54.34
I12	2.22	75.19	71.21
I14	1.38	69.54	78.40
I17	1.92	68.49	64.91
I19	0.00	0.00	0.00
I25	2.48	0.00	2.11
J13	0.24	0.00	0.29
J19	0.04	0.00	0.00
J21	0.00	0.00	0.00
J22	0.00	0.00	0.01
J23	0.00	0.00	0.00
J24	0.00	0.00	0.00
K16	0.50	70.76	10.72
K19	1.77	81.19	14.98
K21	2.32	87.85	54.10
K22	1.51	0.00	7.42
L03	0.44		30.56
L15	1.79	62.60	
L21	1.40		30.93
L27	1.50		46.96
L29	1.40	81.57	17.20

Table S3. Correlation between different antibiotic susceptibilities.

	Pearson	Spearman

		r	p	rho	p
	AMI	0.238	< .001	0.335	< .001
	ATM	0.313	< .001	0.457	< .001
	CAZ	0.302	< .001	0.461	< .001
	CFO	0.428	< .001	0.376	< .001
	CIP	0.340	< .001	0.322	< .001
	CPM	0.307	< .001	0.420	< .001
AMC	CRO	0.379	< .001	0.407	< .001
	CTX	0.353	< .001	0.427	< .001
	ETP	0.280	< .001	0.329	< .001
	GM	0.277	< .001	0.324	< .001
	IMP	0.384	< .001	0.436	< .001
	MER	0.293	< .001	0.340	< .001
	NAL	0.258	< .001	0.380	< .001
	SXT	0.352	< .001	0.542	< .001
	ATM	0.312	< .001	0.620	< .001
	CAZ	0.317	< .001	0.644	< .001
	CFO	0.317	< .001	0.592	< .001
	CIP	0.220	< .001	0.463	< .001
	CPM	0.326	< .001	0.612	< .001
	CRO	0.296	< .001	0.520	< .001
AMI	CTX	0.247	< .001	0.573	< .001
	ETP	0.439	< .001	0.681	< .001
	GM	0.470	< .001	0.780	< .001
	IMP	0.433	< .001	0.502	< .001
	MER	0.477	< .001	0.684	< .001
	NAL	0.103	0.063	0.316	< .001
	SXT	0.091	0.099	0.345	< .001
	CAZ	0.789	< .001	0.772	< .001
	CFO	0.441	< .001	0.677	< .001
	CIP	0.394	< .001	0.506	< .001
	CPM	0.786	< .001	0.814	< .001
	CRO	0.764	< .001	0.774	< .001
ATM	CTX	0.753	< .001	0.776	< .001
	ETP	0.647	< .001	0.747	< .001
	GM	0.371	< .001	0.592	< .001
	IMP	0.406	< .001	0.560	< .001
	MER	0.614	< .001	0.690	< .001
	NAL	0.345	< .001	0.469	< .001
	SXT	0.124*	0.025	0.396	< .001
	CFO	0.445	< .001	0.638	< .001
	CIP	0.337	< .001	0.531	< .001
	CPM	0.712	< .001	0.761	< .001

CAZ

	CRO	0.637	< .001	0.717	< .001
	CTX	0.697	< .001	0.754	< .001
	ETP	0.601	< .001	0.673	< .001
	GM	0.365	< .001	0.627	< .001
	IMP	0.413	< .001	0.582	< .001
	MER	0.582	< .001	0.679	< .001
	NAL	0.273	< .001	0.494	< .001
	SXT	0.139*	0.012	0.446	< .001
CFO	CIP	0.307	< .001	0.463	< .001
	CPM	0.414	< .001	0.649	< .001
	CRO	0.486	< .001	0.637	< .001
	CTX	0.430	< .001	0.657	< .001
	ETP	0.456	< .001	0.612	< .001
	GM	0.400	< .001	0.557	< .001
	IMP	0.454	< .001	0.475	< .001
	MER	0.499	< .001	0.616	< .001
	NAL	0.128*	0.020	0.363	< .001
	SXT	0.080	0.149	0.319	< .001
CIP	CPM	0.451	< .001	0.510	< .001
	CRO	0.501	< .001	0.529	< .001
	CTX	0.426	< .001	0.466	< .001
	ETP	0.297	< .001	0.417	< .001
	GM	0.446	< .001	0.478	< .001
	IMP	0.203	< .001	0.275	< .001
	MER	0.248	< .001	0.397	< .001
	NAL	0.844	< .001	0.753	< .001
	SXT	0.346	< .001	0.432	< .001
CPM	CRO	0.876	< .001	0.783	< .001
	CTX	0.877	< .001	0.802	< .001
	ETP	0.623	< .001	0.679	< .001
	GM	0.455	< .001	0.638	< .001
	IMP	0.416	< .001	0.557	< .001
	MER	0.592	< .001	0.641	< .001
	NAL	0.338	< .001	0.440	< .001
	SXT	0.176**	0.001	0.408	< .001
CRO	CTX	0.884	< .001	0.769	< .001
	ETP	0.524	< .001	0.555	< .001
	GM	0.424	< .001	0.534	< .001
	IMP	0.414	< .001	0.516	< .001
	MER	0.512	< .001	0.541	< .001
	NAL	0.384	< .001	0.464	< .001
	SXT	0.188	< .001	0.387	< .001
CTX	ETP	0.556	< .001	0.673	< .001

	GM	0.373	< .001	0.594	< .001
	IMP	0.352	< .001	0.538	< .001
	MER	0.530	< .001	0.659	< .001
	NAL	0.310	< .001	0.376	< .001
	SXT	0.189	< .001	0.354	< .001
ETP	GM	0.407	< .001	0.570	< .001
	IMP	0.540	< .001	0.573	< .001
	MER	0.753	< .001	0.807	< .001
	NAL	0.174**	0.002	0.323	< .001
	SXT	0.111*	0.044	0.320	< .001
GEN	IMP	0.386	< .001	0.450	< .001
	MER	0.428	< .001	0.553	< .001
	NAL	0.309	< .001	0.340	< .001
	SXT	0.242	< .001	0.380	< .001
IMP	MER	0.692	< .001	0.724	< .001
	NAL	0.092	0.098	0.244	< .001
	SXT	0.086	0.119	0.269	< .001
MER	NAL	0.081	0.141	0.274	< .001
	SXT	0.041	0.459	0.222	< .001
NAL	SXT	0.353	< .001	0.471	< .001

* p < .05, ** p < .01, *** p < .001

Table S4. Minimal inhibitory concentrations (in µg/ml) of ampicillin, ciprofloxacin and kanamycin in selected strains.

Strain	Ampicillin	Ciprofloxacin	Kanamycin
J24	R*	0.003	2
J13	8	0.003	0.5
G20	4	0.03	2
F19	1	0.0015	4
G03	2	0.007	2
A05	2	0.007	2
I04	1	0.007	0.5
E11	4	0.003	1
I12	1	0.003	0.5
I25	2	0.007	2
MC4100	2	0.003	1
BS1154	2	0.003	1

*Strain is resistant to ampicillin.

References

1. Valencia EY, Barros JP, Ferenci T, Spira B. A Broad Continuum of E. coli Traits in Nature Associated with the Trade-off Between Self-preservation and Nutritional Competence. Microbial ecology. 2022 Jan;83:68–82. https://doi.org/10.1007/s00248-021-01751-6.
